# A signaling molecule from intratumor bacteria promotes trastuzumab resistance in breast cancer cells

**DOI:** 10.1073/pnas.2421710122

**Published:** 2025-01-09

**Authors:** Gege Qin, Xiying Shao, Xiaolong Liu, Jiachao Xu, Xiaojia Wang, Wenxi Wang, Lu Gao, Yuxin Liang, Lina Xie, Dan Su, Hongwei Yang, Wei Zhou, Xiaohong Fang

**Affiliations:** ^a^Key Laboratory of Molecular Nanostructure and Nanotechnology, Research/Education Center for Excellence in Molecular Sciences, Institute of Chemistry, Chinese Academy of Sciences, Beijing 100190, China; ^b^Hangzhou Institute of Medicine, Chinese Academy of Sciences, Hangzhou 310022, Zhejiang, China; ^c^Department of Basic Medical Sciences, School of Medicine, Tsinghua-Peking Center for Life Sciences, Institute of Immunology, Beijing Key Lab for Immunological Research on Chronic Diseases, Tsinghua University, Beijing 100084, China; ^d^Department of Breast Medical Oncology, Zhejiang Cancer Hospital, Hangzhou 310022, Zhejiang, China; ^e^University of Chinese Academy of Sciences, Beijing 100049, China; ^f^Key Laboratory of Molecular Developmental Biology, Institute of Genetics and Developmental Biology, Chinese Academy of Sciences, Beijing 100101, China

**Keywords:** intratumor bacteria, targeted therapy, trastuzumab resistance, bacterial signaling

## Abstract

Emerging evidence indicates that intratumor bacteria exist as an active and specific tumor component in many tumor types beyond digestive and respiratory tumors. However, the biological impact and responsible molecules of such local bacteria–tumor direct interaction on cancer therapeutic response remain poorly understood. Trastuzumab is among the most commonly used drugs targeting the receptor tyrosine-protein kinase erbB-2 (ErbB2) in breast cancer, but its resistance is inevitable, severely limiting its clinical effectiveness. Here, we demonstrate that the quorum-sensing signaling molecule N-(3-oxo-dodecanoyl) homoserine lactone (3oc), a chemical compound released by *Pseudomonas aeruginosa* (*P. aeruginosa*), one tumor-resident bacteria with a relative high abundance in breast cancer, promotes breast cancer cell resistance to trastuzumab. Mechanically, 3oc directly leads to spontaneous dimerization of the transforming growth factor β (TGF-β) type II serine/threonine kinase receptor on the cell membrane in a ligand-independent manner. The 3oc-induced TGF-β signaling subsequently triggers ErbB2 phosphorylation and its downstream target activation, overcoming the inhibition effect of trastuzumab on ErbB2. With specific real-time qPCR, fluorescence in situ hybridization imaging, and liquid chromatography ionization tandem mass spectrometry analyses of clinical samples, we confirmed that *P. aeruginosa* and its signaling molecule 3oc exist in breast cancer tissues and there is a clinical correlation between *P. aeruginosa* colonization and trastuzumab resistance. This work expands the biological functions of intratumor bacteria in cancer treatment responsiveness and provides a unique perspective for overcoming trastuzumab resistance.

## Significance

Recent studies have revealed that intratumoral bacteria are active as an emerging tumor component for many unexpected tumor types beyond gastrointestinal cancers but with unclear biological functions. In this study, we provide evidence that signaling molecules from intratumor bacteria participate in cancer resistance to targeted therapy, suggesting a direct effect microbiome could perform on cancer treatment. Our finding offers insights into bacteria-tumor cross talk, providing a valuable reference for optimizing targeted therapeutic strategies.

Growing evidence suggests that the human microbiota is a pivotal regulator in cancer progression, particularly in therapeutic response to immunotherapy and chemotherapy (1, 2). Previous studies on microbiome functions in cancer treatment mainly focused on the indirect effects of gut microbiota on distant tumors, especially the systematic immunomodulatory roles of gut bacteria themselves or their metabolites (3–6). A few studies have found the direct regulation effects of gut microbiota on gastrointestinal cancers through uptaking and inactivating anticancer drugs or promoting cell autophagy and survival (7, 8). Recently, intratumoral bacteria have been identified and characterized inside many unexpected tumors beyond the gastrointestinal and respiratory tract cancers, which were previously considered sterile (9, 10). As an emerging component of the tumor microenvironment, these tumor-resident bacteria can potentially influence tumors on a more local and subtle scale (11). However, the roles of these intratumor bacteria in cancer response to drug treatment remain poorly understood.

Notable from the recent intratumor microbiome analysis, the microbiota in breast tumors is richer than other tumor types but with unclear biological functions (9). For breast carcinoma patients, approximately 20% have copy number amplification of receptor tyrosine-protein kinase erbB-2 (*ERBB2*) gene, resulting in highly expressed ErbB2 protein (12), which is associated with increased tumor aggressiveness, relapse, and mortality (13). Using ErbB2 monoclonal antibodies, typically trastuzumab (14), to block excess ErbB2 on the cell membrane and inhibit aberrant downstream signaling cascades represents the standard therapeutic option for ErbB2-overexpressing breast cancer patients (15). Unfortunately, the response rate of trastuzumab in these patients does not exceed 50% (16). Trastuzumab resistance has been extensively studied with isolated tumor cell lines and immune-deficient mice in aseptic environments. Although promising results have been reported, it remains a foremost clinical obstacle (17). Investigating the molecular mechanism of trastuzumab resistance in a more complex context, especially considering intratumor microbiome, has significant clinical value.

Available 16S rDNA sequencing data have revealed that *Pseudomonas aeruginosa* (*P. aeruginosa*) is one of the prevalent intratumoral bacteria in breast cancer (9). *P. aeruginosa* is an opportunistic pathogen that has been considered for a long time as a leading cause of septicemia in cancer patients (18). Previous works from our group and others have demonstrated that N-(3-oxo-dodecanoyl) homoserine lactone (3oc), a quorum-sensing signaling molecule secreted by *P. aeruginosa*, triggered host immune cell apoptosis (19–22). In this work, we investigated the effect of 3oc on breast cancer cells in their therapeutic response to trastuzumab. We found that the biological function of 3oc on breast cancer cells is different from that on immune cells: 3oc promoted breast cancer cells’ resistance to trastuzumab rather than inducing their death. With single-molecule analysis and biochemical assays, we revealed that 3oc directly increased the dimerization of transforming growth factor β type II serine/threonine kinase receptor (TβRII), thus activating the TGF-β pathway without the presence of its ligand TGF-β1. The 3oc-induced TGF-β signaling promoted the activation of ErbB2 and the downstream pathways of Mitogen-activated protein kinase (MAPK) and phosphoinositide 3-kinase/protein kinase B (PI3K/Akt) for cell proliferation and survival. Our results provided evidence that the intratumor bacteria could directly impact cancer response to targeted therapy, as they can utilize their signaling molecules for direct local interactions with surrounding tumor cells. These findings suggested that intratumor bacteria is an extrinsic tumor factor contributing to trastuzumab resistance in breast cancer. While *P. aeruginosa* is prevalent in many cancer types, we also observed a similar effect of 3oc on other cancer cell lines. This study sheds light on possible patient-to-patient variability of the resident intratumor microbiota in cancer response to existing therapeutic strategies and offers unique opportunities for their clinical treatment.

## Results

### 3oc Desensitized Breast Cancer Cells to Trastuzumab.

**Fig. 1.**
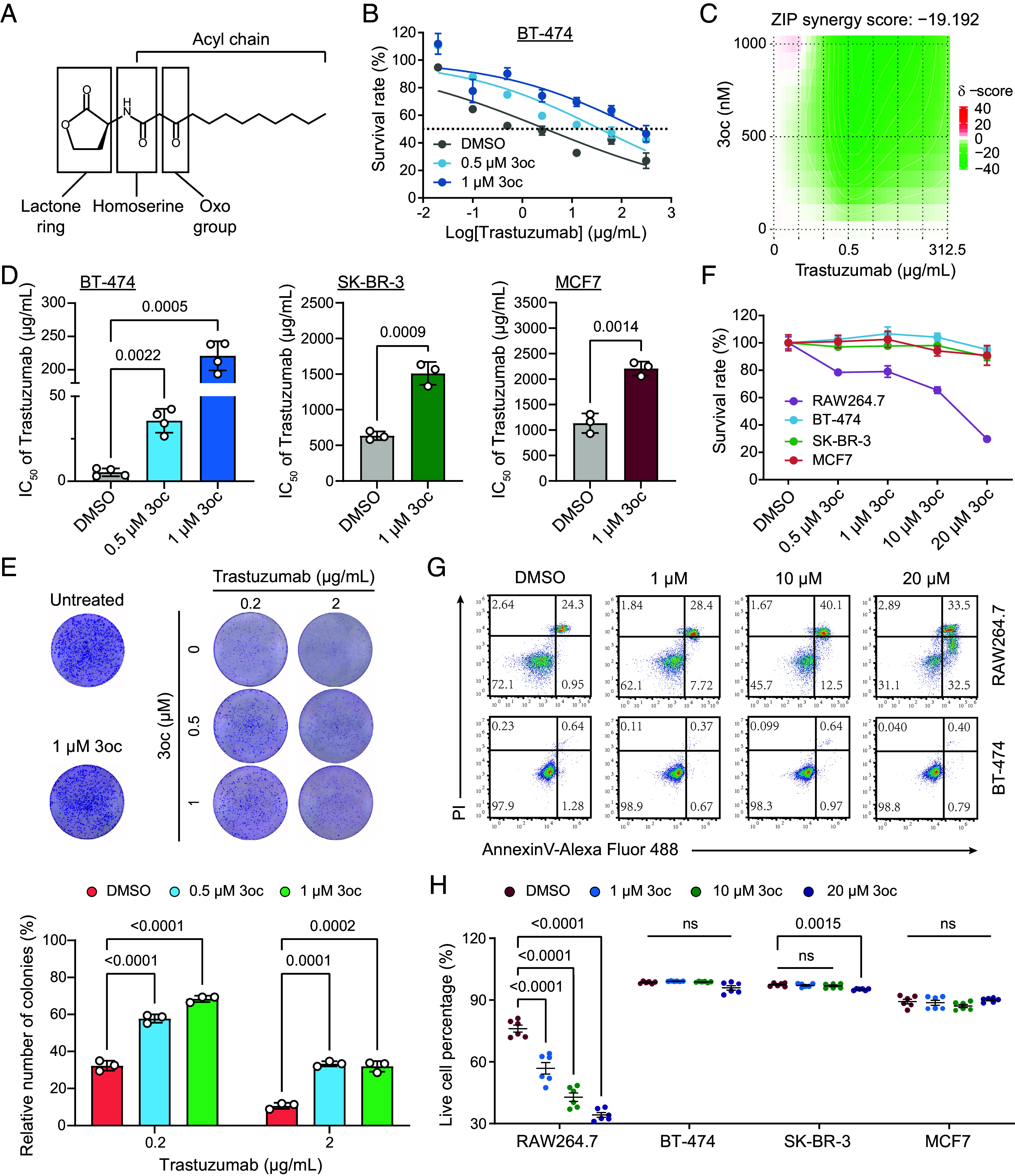
3oc participated in trastuzumab resistance for breast cancer cells. (*A*) The chemical structure of 3oc. (*B*) The dose–response analysis of BT-474 cells to trastuzumab (0 to 312.5 μg/mL) in the presence or absence of 3oc for 72 h. Data are mean ± SD, n = 6 biological replicates. (*C*) Heat maps of combination responses for trastuzumab and 3oc. ZIP Synergy scores <0 indicated antagonism (green regions), and scores <−10 were considered strong antagonism. (*D*) The IC_50_ of trastuzumab in breast cancer cells cotreated with various dosages of 3oc. Cell viability was determined through the Cell Counting Kit 8 (CCK8) assay. For BT-474 cells, the overall *P* value is <0.0001 and η^2^ is 0.985. Cohen’s d is 8.87 for SK-BR-3 cells and 6.45 for MCF7 cells. Data are mean ± SD, n = 3 to 4 independent experiments, each with six biological replicates. (*E*) Representative colony formation images of BT-474 cells treated with the indicated concentrations of trastuzumab and 3oc (*Up* panel). Colony-forming units were determined for each treatment group (*Bottom* panel). The overall *P* value is <0.0001. Data are mean ± SD, n = 3 biological replicates. (*F*) Cell proliferation curves of breast cancer cells treated with 3oc in indicated concentrations for 72 h. Data are mean ± SD, n = 6 biological replicates. (*G* and *H*) Representative flow cytometry images of the annexin-5/PI double staining in cells treated with 3oc (*G*) and the live cell percentage in different groups were further quantified (*H*). Data are mean ± SD, n = 6 biological replicates. The horizontal lines labeled with ns in the bar graph represent the groups with no significant difference compared to the control. Statistical significance was tested by one-way ANOVA with Dunnett’s multiple comparisons test (*D* and *H*), two-tailed unpaired Student’s *t* test (*D*), and two-way ANOVA with Dunnett’s multiple comparisons test (*E*), respectively.

We first examined whether 3oc (molecular structure shown in Fig. 1*A*), secreted from *P. aeruginosa*, participated in developing breast cancer resistance to trastuzumab. Three breast cancer cell lines, BT-474 (ErbB2-overexpression and trastuzumab-sensitive) (23), SK-BR-3 (ErbB2-overexpression and trastuzumab-resistant) (24), and MCF7 (ErbB2-low-expression) (25), were selected (*SI Appendix*, Fig. S1*A*) for trastuzumab treatment. Their expression levels of endogenous ErbB2 were verified by immunoblotting (*SI Appendix*, Fig. S1*B*).

Trastuzumab inhibited cell proliferation for all three cell lines in a dose-dependent manner, and BT-474 cells were relatively more sensitive to trastuzumab than the other cell lines (Fig. 1*B* and *SI Appendix*, Fig. S2 *A* and B). For BT-474 cells, 3oc introduction reduced their sensitivity to trastuzumab (Fig. 1*B*). Specifically, the half maximal inhibitory concentration (IC_50_) value of trastuzumab was 5.3 μg/mL, which increased to 32.6 μg/mL or 220.8 μg/mL in the presence of 0.5 μM or 1 μM 3oc, respectively (Fig. 1*D*), indicating that 3oc acted antagonistically with trastuzumab in inhibiting cell proliferation.

The effect of 3oc on trastuzumab response was further evaluated using the SynergyFinder program to estimate the zero interaction potency (ZIP) synergy scores. The ZIP of −19.192 for BT-474 cells suggested a robust antagonistic effect (Fig. 1*C*, green regions) since ZIP values less than 0 indicated antagonism, and less than −10 were considered strong antagonism (26).

Similar antagonistic effects were also detected in SK-BR-3 and MCF7 cells, with the corresponding IC_50_ of trastuzumab increasing from 635 μg/mL to 1510.3 μg/mL and from 1132.9 μg/mL to 2207.3 μg/mL, respectively in the presence of 1 μM 3oc (Fig. 1*D*). The 3oc’s effect on SK-BR-3 and MCF7 cells was less significant than on BT-474 cells, probably because they were initially quite resistant to trastuzumab. In addition, while both low and high dosages of trastuzumab substantially reduced the number of breast cancer cell colonies, these suppression effects were attenuated by 3oc (Fig. 1*E* and *SI Appendix*, Fig. S2*C*). Our results indicated that 3oc protected breast cancer cells from trastuzumab-induced growth inhibition.

It has been reported that 3oc can mediate immune suppression by inducing cell apoptosis in immune cells (22). We wondered whether the effect of 3oc on trastuzumab response with breast cancer cells is also attributed to the modulation of cell viability. As a positive control group, the viability inhibition of macrophage RAW264.7 cells by 3oc was exceedingly potent, e.g., exposure to 1 μM 3oc was enough to result in 21.0% proliferation inhibition (Fig. 1*F*) and 19.3% apoptosis (Fig. 1*G*). However, all the breast cancer cells showed no noticeable change in cell viability and apoptosis after treatment with 1 μM to even 20 μM 3oc alone (Fig. 1 *F*–*H* and *SI Appendix*, Fig. S3). In contrast, the combination treatment with only 1 μM 3oc clearly increased the IC_50_ of trastuzumab.

A previous report identified major vault protein (MVP) as a potential target of 3oc using normal human bronchial epithelial cells. It revealed that the binding of 3oc to MVP could increase the kinase activity of the protein p38 and attenuate apoptosis in immune cells (21). We compared macrophage RAW264.7 cells with breast cancer BT-474 cells regarding their biochemical features relevant to apoptotic effects of 3oc and MVP–p38 axis activation. A distinguished increase in cleaved caspase-3 was evident in response to 3oc in RAW264.7 cells but imperceptible in BT-474 cells (*SI Appendix*, Fig. S4*A*). Moreover, while 3oc induced enhancement of p38 phosphorylation in RAW264.7 cells, this effect on the phosphorylation status of p38 was not observed in BT-474 cells (*SI Appendix*, Fig. S4*B*). Therefore, our results suggested that 3oc enabled trastuzumab resistance for breast cancer cells, and this impact could not be attributed to the MVP–p38 axis in cell apoptosis, thus different from previous work with immune cells.

### 3oc Activated the TGF-β and ErbB2 Pathway in Breast Cancer Cells.

**Fig. 2.**
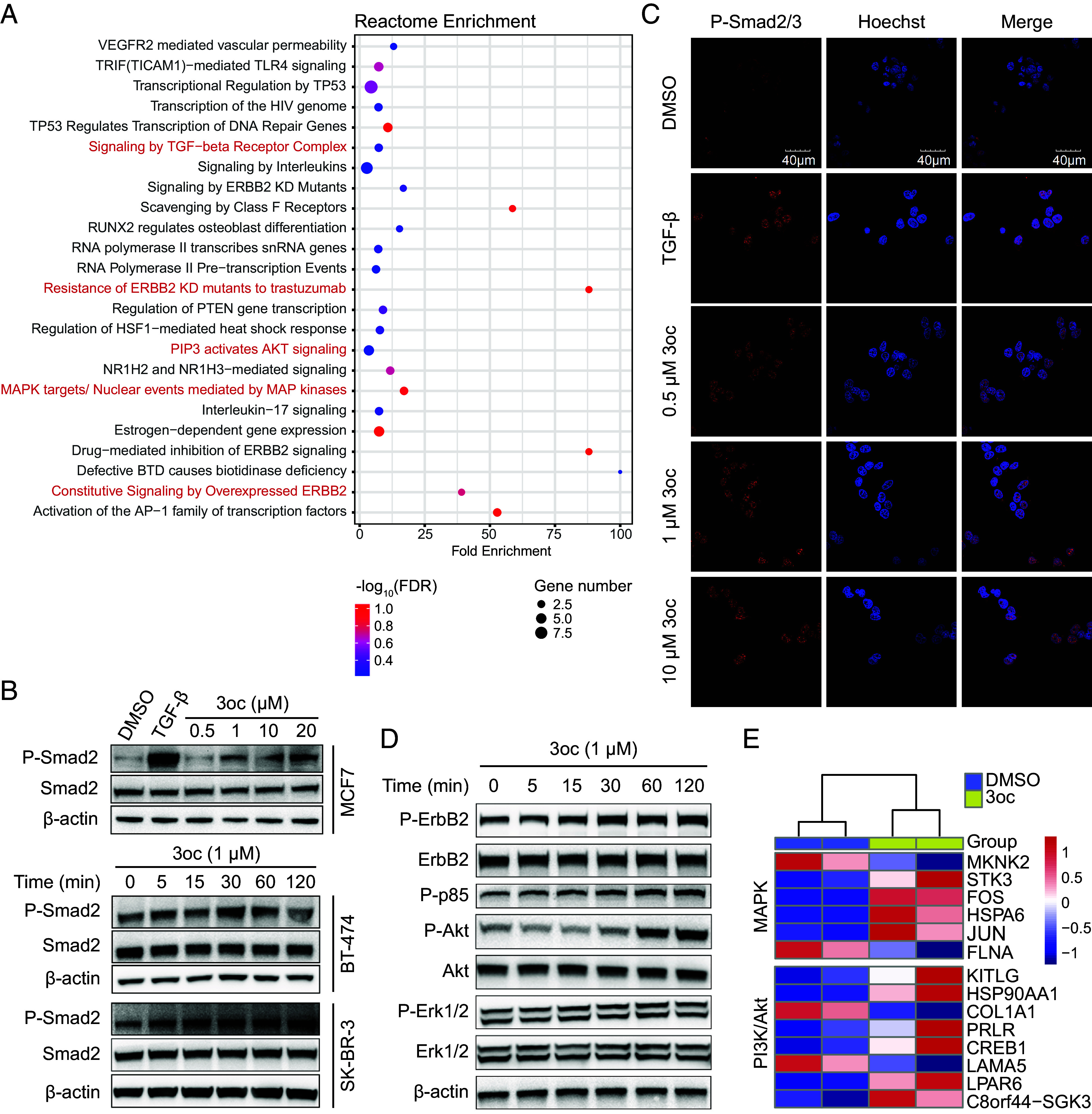
3oc activated TGF-β and ErbB2 signaling in breast cancer cells. (*A*) Reactome gene sets enrichment analysis of the differentially expressed genes in BT-474 cells before and after 3oc treatment (1 μM) for 45 min. Dot plot sizes indicate the gene counts enriched in the term or signaling pathway. The color of each dot represents the degree of significance. (*B*) Representative western blot of phosphorylated Smad2 in MCF7 cells stimulated with 10 ng/mL TGF-β1 or with 3oc in indicated concentrations (*Upper* panel) and in BT-474 and SK-BR-3 cells stimulated with 1 μM 3oc in time gradient (*Bottom* panel). (*C*) Immunofluorescence imaging of p-Smad2/3 (mAb, Alexa-647) and cell nuclei (Hoechst 33258) by confocal microscopy in BT-474 cells stimulated with 10 ng/mL TGF-β1 or 3oc at the indicated concentrations. (*D*) Representative western blot images of phosphorylated ErbB2, p85, Erk, and Akt in BT-474 cells stimulated with 1 μM 3oc in time gradient. (*E*) Heat map depicts the differential abundance of MAPK and PI3K pathway-associated mRNA in BT-474 cells upon 3oc treatment or left untreated, each with two biological repeats. Blue and red indicate down and upregulation, respectively. The image is representative of 3 independent experiments (*B* and *D*).

We then moved to investigate the molecular mechanism of 3oc-induced trastuzumab resistance in breast cancer cells. An mRNA-seq analysis was conducted before and after 3oc treatment, and a core gene expression signature consisting of 169 differentially expressed genes was identified (false discovery rate < 0.02 and fold change ≥ 1.5) (*SI Appendix*, Fig. S5 *A* and B and Table S1). Gene Sets Enrichment Analysis based on the Reactome database revealed that these differentially expressed genes were primarily enriched in the TGF-β and ErbB2-associated pathways (e.g., Resistance of ErbB2 KD mutants to trastuzumab, Constitutive Signaling by Overexpressed ErbB2, Signaling by TGF-beta Receptor Complex and so on) (Fig. 2*A* and *SI Appendix*, Table S2). This conclusion was also supported by Gene Sets Enrichment Analysis results based on other databases (*SI Appendix*, Fig. S5 *C* and D and Tables S3 and S4). Trastuzumab binds directly to ErbB2, inhibiting ErbB2 phosphorylation and down-regulating the constitutive signaling of downstream pathways. TGF-β signaling could functionally synergize with the ErbB2 pathway network in various ways to promote trastuzumab resistance (27–29). Therefore, we speculated that 3oc might promote trastuzumab resistance by reactivating the ErbB2 pathway directly or through the TGF-β signaling.

The TGF-β signaling is propagated by the signal transducers Smad2/Smad3, which are activated by transphosphorylation of a heteromeric complex of TβRI and TβRII on the cell membrane. The activated Smad2/Smad3 then translocates into the nucleus to regulate related gene expression (30). Our western blot results showed that 3oc treatment led to a substantial increase of phosphorylated Smad2 at Ser465/467 in a time-dependent manner, which occurred within 5 min after 3oc addition (Fig. 2*B*). Immunofluorescence imaging revealed an enhanced fluorescent signal of phosphorylated Smad2/Smad3 in breast cancer cells after 3oc treatment (Fig. 2*C* and *SI Appendix*, Fig. S6*A*), and these fluorescent signals were mainly located in the nucleus. Both western blot and immunofluorescence experiments demonstrated that 3oc activated the TGF-β signaling in breast cancer cells.

Similarly, we also checked the activation of the ErbB2 pathway after 3oc treatment in breast cancer cells. Western blotting revealed an increased phosphorylation level of ErbB2 and its core downstream targets, including PI3K regulatory subunit p85-alpha (p85), mitogen-activated protein kinase 3/1 (Erk1/2), and Akt (Fig. 2*D*). In line with this, our single sample gene set enrichment analysis (ssGSEA) also showed 3oc up-regulated the expression of many proteins involved in the MAPK and PI3K/Akt pathways (Fig. 2*E* and *SI Appendix*, Tables S5 and S6). MAPK and PI3K/Akt pathways are interlinked in TGF-β and ErbB2 signaling transduction and have been reported to promote trastuzumab resistance in breast cancer models and primary malignancies (31–33). Therefore, our results of mRNA sequencing, immunofluorescence imaging, and immunoblotting suggested that 3oc activated the TGF-β and ErbB2 pathways in breast cancer cells, which rendered breast cancer cells insensitive to trastuzumab.

### 3oc Promoted Trastuzumab Resistance by Directly Activating the TGF-β Pathway.

Our above western blot results showed that the 3oc-induced activation of the TGF-β pathway was earlier than the ErbB2 pathway. More specifically, 3oc required at least 15 to 30 min to induce a visible increase of phosphorylated ErbB2, p85, and Erk1/2, and at least 60 min to lead to a similar increasing tendency in Akt phosphorylation (Fig. 2*D* and *SI Appendix*, Fig. S6*B*). However, the Smad2 activation could be observed as soon as 5 min after 3oc addition (Fig. 2*B*). Published works have proved that TGF-β signaling potentiates oncogenic ErbB2 signaling and participates in trastuzumab resistance (27, 28). Based on this, we expected that 3oc induced ErbB2 activation and trastuzumab resistance in breast cancer cells via activating the TGF-β signaling pathway.

**Fig. 3.**
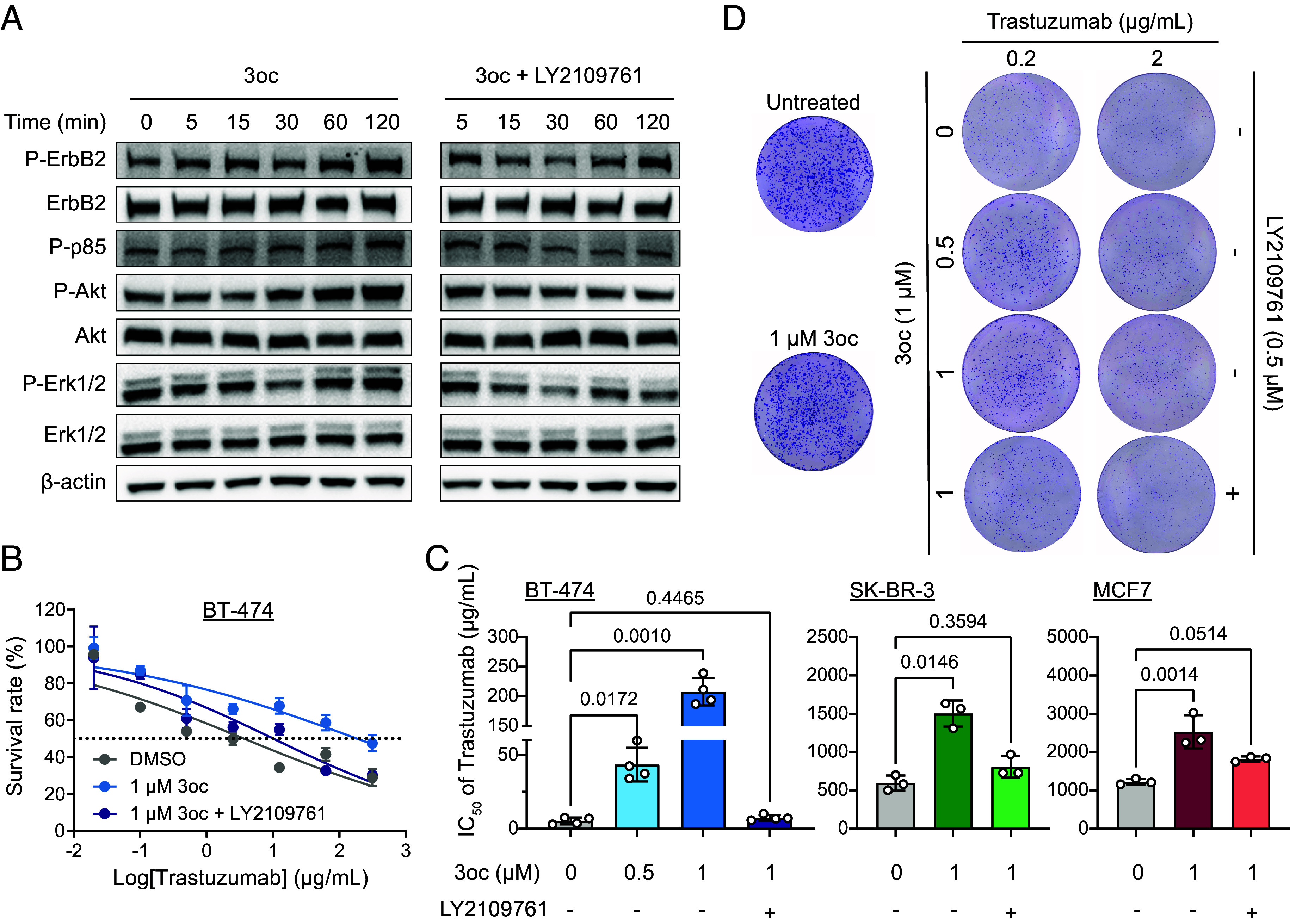
3oc promoted trastuzumab resistance in breast cancer cells via the TGF-β pathway. (*A*) Representative western blot images of phosphorylated ErbB2, P85, Erk, and Akt in BT-474 cells stimulated with 3oc (1 μM) or the combination of 3oc (1 μM) and LY2109761 (0.5 μM) in time gradient. (*B*) The dose–response analysis of BT474 cells against trastuzumab (0 to 312.5 μg/mL) after the treatment of indicated compounds for 72 h. (*C*) The IC_50_ values of breast cancer cells in response to trastuzumab when treated with 3oc alone or combined with LY2109761 (0.5 μM) for 72 h. Data are the mean ± SD, n = 3 to 4 independent experiments, each with six biological replicates. (*D*) Representative colony formation images of BT-474 cells treated with the indicated concentrations of trastuzumab in the presence or absence of 3oc or 3oc + LY2109761. The image is representative of 3 independent experiments. Statistical significance was tested by one-way ANOVA with Dunnett’s multiple comparisons test in *C*. The overall *P* values for the one-way ANOVA test are <0.0001 (BT-474), 0.0036 (SK-BR-3), and 0.0024 (MCF7).

The addition of LY2109761, a small-molecule inhibitor of TβRII/TβRI, abrogated the 3oc-induced Smad2 phosphorylation in BT-474 cells (*SI Appendix*, Fig. S7*A*), suggesting an effective blockade of TGF-β signaling in the context of 3oc treatment. The 3oc-induced phosphorylation of ErbB2 and its downstream targets, ErbB2, p85, Akt, and Erk, were all reversed by further LY2109761 treatment (Fig. 3*A*). These results suggested that 3oc aroused ErbB2 pathway activation via TGF-β signaling. The cell proliferation assay showed that the 3oc increased IC_50_ value of trastuzumab in breast cancer cells was also reduced to the background level by LY2109761 (Fig. 3 *B* and *C* and *SI Appendix*, Fig. S7 *B* and C). Moreover, while 3oc enhanced the colony formation efficiency of trastuzumab-treated BT-474 cells, this effect was also attenuated by LY2109761 addition (Fig. 3*D* and *SI Appendix*, Fig. S7*D*). The above results suggested that 3oc-activated ErbB2 signaling and trastuzumab resistance were mainly driven by TGF-β pathway activation.

### 3oc Activated the TGF-β Pathway by Directly Triggering TβRII Autodimerization in a Ligand-Independent Manner.

**Fig. 4.**
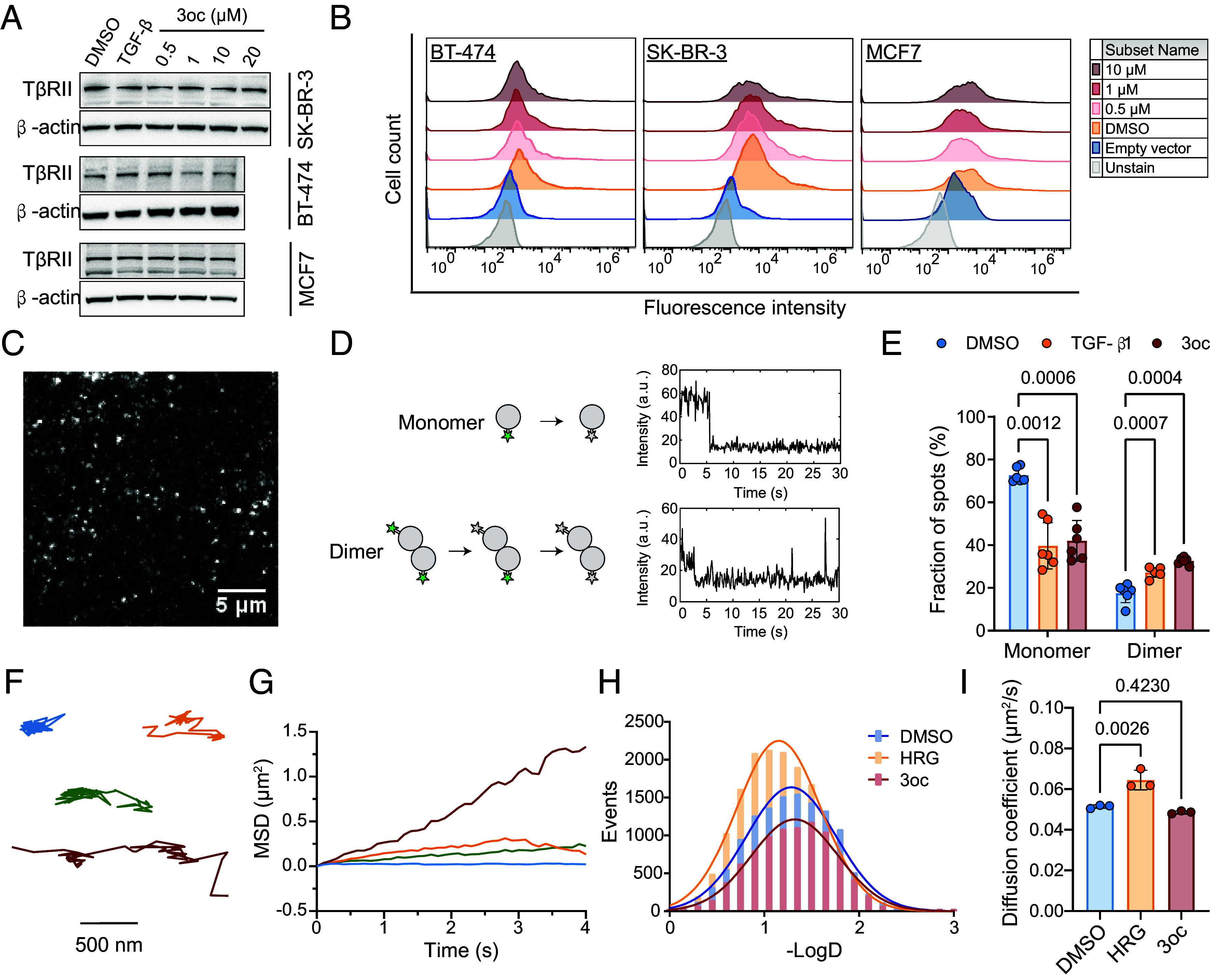
3oc increased TβRII dimerization without changing TβRII expression level. (*A*) Representative western blot of the cellular expression levels of TβRII in breast cancer cells treated with 10 ng/mL TGF-β1 or with a 3oc concentration gradient for 5 min. (*B*) Representative plots for the cell-surface expression of HA-TβRII in breast cancer cells treated with the indicated concentrations 3oc for 5 min. (*C*) A typical single-molecule image shows the distribution of TβRII-EGFP molecules with different oligomerization states at the plasma membrane of an MCF7 cell. The image is a section (26 × 26 μm) of the first frame from a raw movie after background subtraction. (*D*) Illustration of the single-molecule photobleaching step-counting assay (*Left*). Two representative plots of fluorescent intensity versus time of EGFP emission in TβRII-EGFP with different aggregation states showing one- and two-step bleaching. The *x*-axis represents illumination time; the *y*-axis represents an arbitrary unit (a.u.) of emission intensity. (*E*) The frequency of the one- and two-step bleaching events for TβRII-EGFP in MCF7 cells stimulated with 10 ng/mL TGF-β1 or with 1 μM 3oc for 5 min. Data are the mean ± SD, n = 6 independent experiments. The overall *P* value is 0.0012. The results of each experiment were counted from 10 to 40 cells with more than 2,000 individual fluorescent molecules. (*F*) Illustrative trajectories of single ErbB2 molecules with different diffusion coefficients. (*G*) The plot of MSD versus time for receptors with trajectories shown in *F*. (*H*) Distributions of the diffusion coefficient of membrane-docked ErbB2-EGFP molecules in resting MCF7 cells (n = 15 cells) or MCF7 cells stimulated with 10 ng/mL HRG (n = 10 cells) or 1 μM 3oc (n = 11 cells) for 5 min. (*I*) The most probable D values of ErbB2-EGFP molecules from MCF7 cells under different conditions as set in *H*. Data are the mean ± SD, n = 3 independent experiments. The overall *P* value is 0.0011. Three distribution histograms of the diffusion coefficient were acquired through three independent experiments, each representing the data from 5,000 to 10,000 trajectories of individual ErbB2 molecules in 10 to 20 cells. Fitting each histogram with Gaussian distribution yielded the most probable D values for each treatment group. The image is representative of at least three independent experiments (*A* and *B*). Statistical significance was tested by two-way ANOVA with Dunnett’s multiple comparisons test (*E*) and one-way ANOVA with Dunnett’s multiple comparisons test (*I*), respectively.

We then explored the molecular mechanism responsible for 3oc-induced TGF-β pathway activation. The TGF-β signaling cascade is triggered by the dimerization of membrane receptor TβRII upon ligand stimulation, which facilitates the phosphorylation of TβRII/TβRI and activation of downstream pathways (34, 35). Western blot analysis showed no detectable increase in the total TβRII expression level in the presence of different concentrations of 3oc (Fig. 4*A*). We also analyzed the amount of membrane-located TβRII in 3oc-treated breast cancer cells by flow cytometry (Fig. 4*B*). The fluorescence intensity distribution also showed no distinct shift after treatment with different concentrations of 3oc. These results excluded the possibility that 3oc treatment led to either overexpression or membrane-recruitment of TβRII for constitutive TGF-β signaling. Moreover, mRNA sequencing results demonstrated that 3oc treatment did not affect the expression level of TGF-β1, the TβRII ligand, indicating the 3oc-activated TGF-β signaling was also ligand-independent (*SI Appendix*, Fig. S8*A*).

We then asked whether 3oc directly triggered TβRII autodimerization. To see the effect of 3oc on the cell membrane, we adopted a single-molecule imaging technique to explore the molecular behavior of green fluorescent protein (EGFP) tagged TβRII at the plasma membrane of breast cancer cells (36, 37). Most TβRII-EGFP molecules appeared as well-dispersed diffraction-limited fluorescent spots on the cell surface (Fig. 4*C* and *SI Appendix*, Fig. S8 *B* and C). We counted the photobleaching steps of TβRII-EGFP single molecules to study their stoichiometry (Fig. 4*D* and Movie S1). The majority of spots showed single-step photobleaching traces in the resting cells, suggesting TβRII mainly exists as a monomer in the absence of ligand (Fig. 4*E*). At 5 min after 3oc addition, the proportion of the two-step photobleaching fluorescent spots increased significantly, confirming that TβRII dimerization was triggered rapidly (Fig. 4*E* and *SI Appendix*, Fig. S8*D*). Similar enhanced dimerization of TβRII typically requires binding of TGF-β ligand to TβRII (Fig. 4*E*). However, no ligands were introduced in our setting of 3oc-treated breast cancer cells.

Our results from the cellular thermal shift assay (CETSA) showed that the addition of 3oc did not affect the thermal stability of either TβRII or ErbB2 (*SI Appendix*, Fig. S9), suggesting that 3oc did not engage with these proteins in breast tumor cells. Furthermore, we reanalyzed the published chemical proteomic results from the 3oc derivatives-treated normal human bronchial epithelial cells (21). Among the 1,285 potential 3oc target peptides, we have failed to find any of our described proteins involved in TβRII/ErbB2 signaling. According to our previous finding, the long acyl chain of 3oc incorporated into the cell membrane and altered the lipid-raft structure, resulting in tumor necrosis factor receptor 1 trimerization and spontaneous signal initiation for immune cells (22). Therefore, for breast cancer cells, we expected that 3oc probably acted similarly on lipid-raft, resulting in TβRII autodimerization and TGF-β signaling transduction.

ErbB2 functions mainly through heterodimerization of EGFR and ErbB3 without forming ligand-induced homodimers (38). We thus evaluated the diffusion dynamics, rather than stoichiometry, of ErbB2-EGFP at the cell membrane through a live-cell single-molecule tracking method to assess its activated status after 3oc treatment (Fig. 4*F*). The mean square displacement (MSD) change with time was plotted to obtain the diffusion coefficient (D) for each ErbB2-EGFP trajectory (Fig. 4*G* and Movies S2 and S3) (39). According to our previous study, ErbB2 diffused apparently faster upon activation by heregulin β1 (HRG), an ErbB ligand (40). Therefore, we set up the same treatment as a positive control to indicate the activation of ErbB2 and got consistent results that HRG stimulation shifted the diffusion coefficient of ErbB2-EGFP molecules toward higher values, from 0.0514 ± 0.0007 μm^2^/s in resting cells to 0.0644 ± 0.0040 μm^2^/s in HRG-stimulated cells (Fig. 4*H*). In contrast, 3oc treatment did not alter the diffusion dynamics of ErbB2, with the diffusion coefficient maintained at 0.0486 ± 0.0006 μm^2^/s (Fig. 4*I*). Therefore, 3oc did not directly alter the membrane dynamic behaviors of ErbB2 to activate its downstream pathway.

**Fig. 5.**
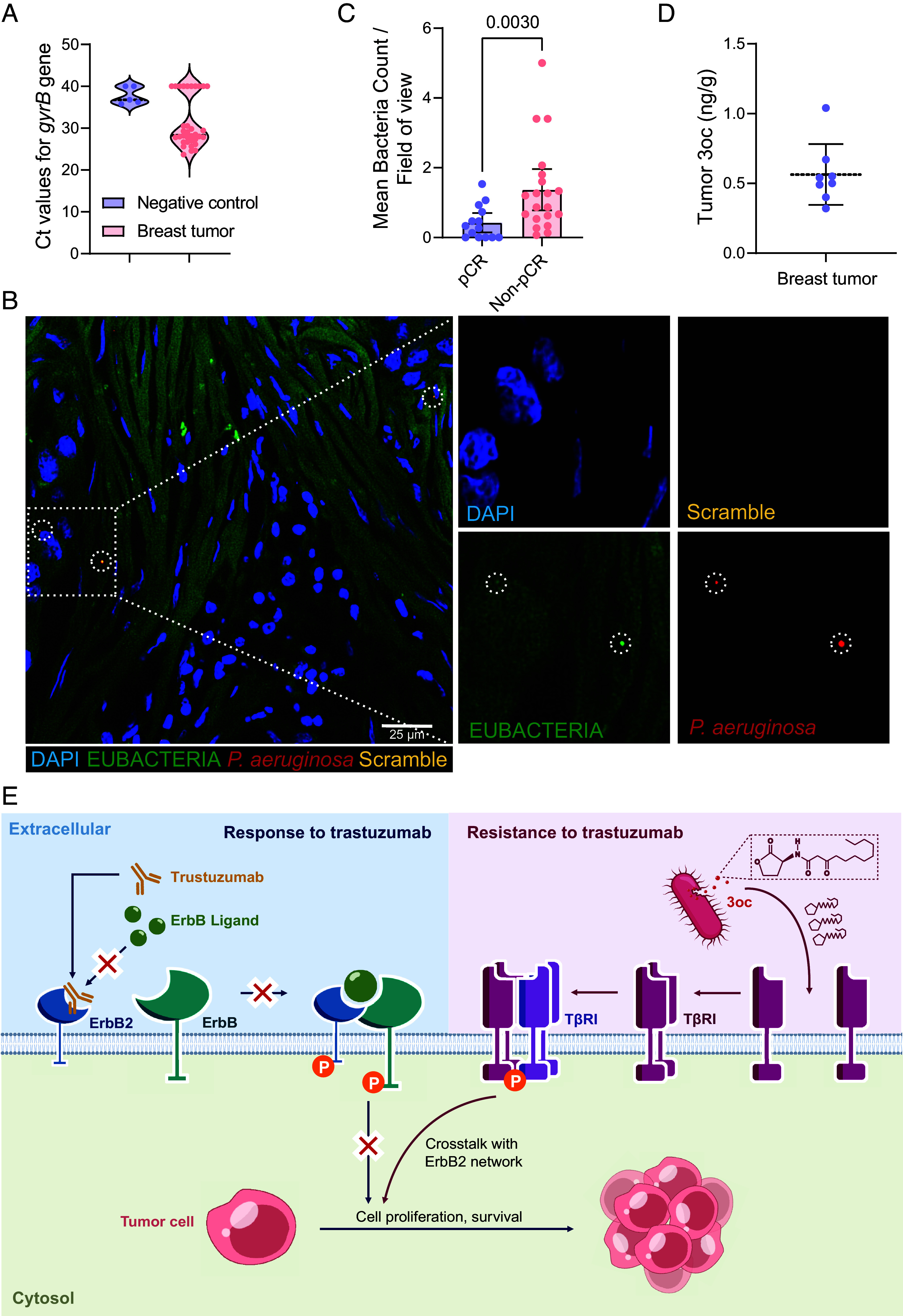
Detection of *P. aeruginosa* and 3oc in human breast cancer tissues and their function. (*A*) The Ct values of the *gyrB* probe for *P. aeruginosa* detection using genomic DNA extracted from FFPE sections of human breast carcinoma tissues (n = 34). (*B*) Representative FISH staining images of *P. aeruginosa* in one human breast tumor sample. Tissue sections were hybridized with eubacteria probes (green), *P. aeruginosa* probes (red), and scramble probes (yellow). *P. aeruginosa* is encircled by white dashed circles. Boxed regions are enlarged and shown. (*C*) Mean bacteria count inside tumor tissues from breast cancer patients with or without pathological complete remission after trastuzumab treatment (n = 14 and 20, respectively). Each data point represents one sample. Data are represented as median and 95% CI. Cohen’s d = 1.02. (*D*) The quantities of 3oc assessed by LC–ESI–MS/MS in frozen human breast tumor samples that tested positive. Data are mean ± SD. (*E*) Proposed mechanism for the relationship of 3oc secreted by *P. aeruginosa* with trastuzumab-resistance. Statistical significance was tested by the nonparametric Mann–Whitney test (*C*).

Taken together, our results indicated that 3oc acted on breast cancer cells by directly triggering a higher extent of spontaneous dimerization of TβRII rather than altering their total protein level or subcellular localization. This led to TGF-β pathway activation, subsequently synergizing with the ErbB2 signaling network, and consequently promoted resistance to the anti-ErbB2 drug trastuzumab (Fig. 5*E*).

### Clinical Relevance of Intratumor Infection with *P. aeruginosa* in Breast Carcinoma Patients.

3oc is one of the major quorum-sensing signaling molecules of *P. aeruginosa*. We then evaluated whether *P. aeruginosa* and 3oc were detectable in the breast tumors of patients to determine their clinical significance. We extracted genomic DNA from formalin-fixed paraffin-embedded (FFPE) sections of breast carcinoma tissues (n = 34) for real-time qPCR. Specific primers and a TaqMan probe targeting the DNA gyrase subunit B gene *gyrB* of *P. aeruginosa* were used (*SI Appendix*, Fig. S10*A*) (41). The household gene β-globin served as an internal reference gene to eliminate the false negatives introduced by the possible DNA degradation in paraffin samples (42). The specificity of primers and probes for *gyrB* and β-globin have been confirmed using genomic DNA extracted from *P. aeruginosa* and human tissues as templates before the experiment (*SI Appendix*, Fig. S10*B*).

In our cohort of patients, all 34 samples showed a trustworthy amplification signal for β-globin, indicating that the DNAs in these samples were well preserved. We observed that 7 specimens showed positive *P. aeruginosa* detection, with the cycle threshold (Ct) values of the *gyrB* gene ranging from 25.59 to 29.06. In contrast, the Ct values of negative controls (including DNA extraction controls, no-template PCR amplification controls, and empty paraffin controls) were all higher than 35. This excluded the possibility of laboratory-born contamination (Fig. 5*A* and *SI Appendix*, Fig. S10*C*). The detectable rate of intratumor *P. aeruginosa* in our cohort of breast carcinoma patients is 20.6% (*SI Appendix*, Fig. S10*D*), similar to the 16S sequencing results in the reported intratumor microbiome project (9, 43).

Moreover, we performed fluorescence in situ hybridization (FISH) imaging to visualize and quantify the *P. aeruginosa* in tumor tissues collected from ErbB2-overexpressed breast cancer patients who received trastuzumab treatment. We first validated the specificity and effectiveness of our FISH probe system using *P. aeruginosa*-coated glass slides and mouse mammary tumors injected intratumorally with *P. aeruginosa* (*SI Appendix*, Fig. S11). Subsequently, we analyzed clinical breast tumor specimens using these validated probes. The 16S rRNA/rDNA of *P. aeruginosa* could be frequently detected in these tumor samples (Fig. 5*B*). The *P. aeruginosa* signal was found not only colonized intracellularly but localized intercellularly in the sites distant from the nucleus (*SI Appendix*, Fig. S12). The statistical results showed that the *P. aeruginosa* abundance was significantly higher in patients with a non-pCR (nonpathological complete remission) after trastuzumab management than those who achieved a pCR (Fig. 5*C*). The relatively high amount of *P. aeruginosa* may potentially promote breast cancer resistance to trastuzumab. These results established a clinical correlation between *P. aeruginosa* colonization and trastuzumab resistance.

We also performed liquid chromatography ionization tandem mass spectrometry (LC-ESI-MS/MS) analysis to quantitatively analyze 3oc in frozen tumor tissues collected from breast cancer patients (*SI Appendix*, Fig. S13*A*). Specifically, 3oc was detectable in 8 out of 24 specimens (*SI Appendix*, Fig. S13 *B* and C). The amounts of 3oc in these positive samples were estimated to be in the nanogram per gram (ng/g) range (Fig. 5*D*). Since our FISH imaging results on breast tumor samples indicated the distribution of *P. aeruginosa* is uneven, which is focally enriched, we used the volume percentage data to calibrate, and the results showed that the local concentration of 3oc could reach the μM range (*SI Appendix*, Fig. S13 *D* and E). This concentration is comparable to that we used in our in vitro experiments. Moreover, the poor solubility of 3oc in aqueous solutions (<0.1 mg/mL) also facilitated its accumulation near cellular membranes (22), potentially leading to a high concentration of 3oc to exert its effect on trastuzumab resistance in breast cancer. Previous reports also suggest that in vivo, 3oc concentration can reach hundreds of μM at the site of infection and form a diffusion gradient (44), ending with a concentration of several μM measured in most settings (45).

Our studies with clinical samples not only confirm that intratumoral *P. aeruginosa* exist in ErbB2-positive breast cancers and are active in secreting the signaling molecule 3oc but also underscore the potential clinical relevance of *P. aeruginosa* in modulating the response to trastuzumab treatment.

## Discussion

With the emerging next-generation gene sequencing, tumor-resident bacteria have been comprehensively profiled across much broader cancer types beyond digestive and respiratory tumors, while the biological roles of these intratumor bacteria in cancer treatment remain largely unknown. In this work, through single-molecule imaging as well as biochemical, transcriptomic, bioinformatic, and clinical studies, we demonstrated that the signaling molecule 3oc, produced by *P. aeruginosa*, restricted the response of breast cancer cells to trastuzumab therapy. We revealed that the effect of 3oc in trastuzumab response was aroused via directly triggering TβRII autodimerization on the cell membrane and TGF-β signaling, which in turn synergized with the ErbB2 signaling to activate interlinked downstream targets. Our study provided connection between intratumor bacteria and targeted therapy resistance, suggesting a direct effect of the microbiome on cancer treatment.

The bacterial signaling molecule 3oc has been reported to mediate immune suppression by triggering immune cell apoptosis (22). In this work, we found that the primary biological function of 3oc in breast cancer cells was promoting trastuzumab resistance through the TGF-β/ErbB2 signaling network, with no significant effect on cell apoptosis. As a supplement to the reported mechanisms associated with bacteria-induced resistance to cancer treatments [including metabolizing drugs into an inactive form, inducting tumor cell autophagy to confer chemotherapy resistance, and so on (7, 8)], our work proposed a different mechanism. Bacteria may utilize its signaling chemicals to antagonistically activate specific signaling pathways in tumor cells that have been masked by targeted drugs. Investigating the complex interactions between breast tumor-resident bacteria and cancer cells could yield valuable insight into the specific way individual cancer patients might respond to targeted therapy.

According to previous works, trastuzumab resistance is mainly attributed to genetic and epigenetic alterations in cancer that are responsible for the aberrant constitutive activation of the ErbB2 pathway (16). Nevertheless, emerging evidence suggests that cancer response to this drug is not only determined by intrinsic genetic factors (46). Recent research with mouse models and ErbB2-positive breast cancer patients has shown that antibiotic administration substantially altered the gut microbiota composition and impaired immune-mediated trastuzumab therapeutic activity, revealing the involvement of the gut microbiota in trastuzumab responsiveness (47). Here, we provide evidence that intratumor bacteria *P. aeruginosa* may act as an extrinsic tumor factor to prevent breast cancer cells from trastuzumab-induced proliferation inhibition. This raised a critical clinical insight that the proper implementation of antibiotics to intercept certain bacterial infections, rather than reducing the general diversity of the host microbiota, may potentially facilitate interventions of trastuzumab therapy resistance. Furthermore, besides breast carcinoma, trastuzumab therapy has also been applied to other ErbB2-positive cancers, such as gastric, gastroesophageal junction, and lung cancer (48, 49). We also observed the 3oc-induced TGF-β/ErbB2 network activation and trastuzumab resistance in gastric cancer cells, suggesting that the results we obtained may be a general phenomenon (*SI Appendix*, Figs. S14–S17). Recent studies have shown that the administration of an antibiotic cocktail could free a tumor from resident bacteria (4). Therefore, the development of antibiotic therapy that eliminates *P. aeruginosa* in breast cancer could be considered for future research.

Activation of TGF-β signaling typically requires engagement of TGF-β receptor family members, such as TβRII, with its ligand TGF-β. Several studies have found that some chemical compounds can modulate TGF-β pathway activation in the absence of ligands through up-regulating cell-surface located or total levels of the TβRII (24, 50). Then, the accumulation of excess TβRII at the cell membrane results in constitutive receptor activation due to receptor self-association. In this work, the signaling molecule 3oc secreted by bacteria directly led to spontaneous dimerization of TβRII without using external ligands nor enhancing TβRII expression. These findings suggest a mechanism for the non-ligand-dependent activation mode of the TGF-β pathway.

In the future, the association between the intratumoral levels of *P. aeruginosa* and patient outcomes in trastuzumab therapy will be further explored, which is expected to gain deeper insights into the role of tumor-resident bacteria in clinic cancer therapeutics.

## Materials and Methods

A summary of the methods is reported below, and additional details are referred to *SI Appendix*.

### Cell Culture.

HeLa cells were from Peking Union Medical College. MCF7 cells were a gift from Prof. Kangmin He. BT-474, SK-BR-3, NCI-N87, and H1299 cells were purchased from the American Type Culture Collection (ATCC). RAW264.7 cells were a gift from Prof. Yan Shi of Tsinghua University, Beijing, China. Hela, SK-BR-3, and MCF7 cells were cultured in Dulbecco’s modified Eagle’s medium (DMEM, Gibco) supplemented with 10% fetal bovine serum (FBS, Gibco) and penicillin/streptomycin (100 IU mL-1, Gibco). RAW264.7, BT-474, NCI-N87, and H1299 cells were cultured in RPMI-1640 media (Gibco) supplemented with 10% FBS and penicillin/streptomycin (100 IU mL-1). These cells were grown under standard culture conditions (37 °C, 5% CO_2_ humidified atmosphere). All cell lines used in this work have been authenticated using STR DNA fingerprinting and are free of mycoplasma contamination.

### Immunofluorescence.

BT-474, MCF7, and HeLa cells were plated in cover glass bottom dishes (ø35 mm, Cellvis, California), serum-starved overnight, and allowed to reach 60 to 80% confluence at the time of stimulation. For immunofluorescent staining of intracellular P-Smad2/3, cells were permeabilized with PBS containing 0.5% Triton X-100 and 3 % paraformaldehyde for 10 min and fixed with 4% paraformaldehyde in PBS for 20 min at room temperature. After thorough washing with PBS, the fixed cells were incubated in the blocking buffer (PBS, 0.5% bovine serum albumin) for 30 min, labeled with the diluted primary antibodies (phospho-Smad2/3 rabbit mAb, 1:100) in blocking buffer overnight at 4 °C. The samples were washed three times for 10 min each and then stained with the diluted fluorescent conjugated secondary antibodies (goat anti-rabbit secondary antibody Alexa Fluor Plus 647, 1:200) in blocking buffer for 1 h at 4 °C and washed three times. Before imaging, nuclei were counterstained with Hoechst 33258 (1:500).

The prepared cell samples were observed and recorded under a confocal microscope (FluoView FV1000-IX81, Olympus, Japan) fitted with a 100X (NA 1.40) oil immersion objective. For multicolor imaging, Hoechst was excited by a 405 nm laser (FV5-LD405-2, Olympus), and the emission was collected using a 425 to 475 nm bandpass filter. Alexa Fluor 647 was excited with a 635 nm laser (FV10-LD635, Olympus), and the emission was collected using a 655 to 755 nm band-pass filter. The fluorophores were excited by the corresponding emission filters sequentially. In this setup and under our experiment condition, the color bleed-through between different channels is negligible.

### Single-Molecule Fluorescence Imaging.

To detect individual TβRII or ErbB2 on the cell membrane, the TβRII-EGFP or ErbB2-EGFP plasmid was transfected into MCF7 cells in cover glass bottom dishes and cultured for another 5 to 6 h. Then, MCF7 monolayers (75% confluent) previously serum-starved overnight were stimulated with 3oc, TGF-β, or left untreated for 5 min. Samples were then washed twice with PBS, fixed with 3% paraformaldehyde and 0.1% glutaraldehyde, placed in phenol red-free DMEM, and loaded for imaging. Single-molecule fluorescence imaging was conducted using a homemade objective-type total internal reflection fluorescence microscope (TIRFM) based on Olympus IX71 inverted microscope. The fluorophores (EGFP) were excited by a sapphire 488 nm laser (Coherent). The typical excitation power measured after the laser passed through the objective (an oil immersion objective 100×, 1.45 NA, Olympus) in epi-mode was fixed to 1 mW. The fluorescent signal was collected by the same objective, separated by the dual-view assembly (Optical Insights), and properly filtered with a band-pass filter (525/50 for EGFP) before being projected onto an electron-multiplexing charge-coupled camera (EMCCD, Andor Technology DU-897D-BV). The notch filter (NF01-488, Semrock) was also applied to reject scattered laser light. The electron-multiplying gain of the EMCCD was set to 300. Movies of 300 frames were acquired for each sample at a frame rate of 10 Hz. Andor IQ software and *z*-axis negative feedback equipment (MFC-2000) were adopted to control image acquisition. The photobleaching steps of single molecules at the cell membrane were analyzed according to the method we reported previously (34). In brief, the background fluorescence was subtracted from each frame using the rolling ball method in ImageJ software. Then regions of interest in the movies were extracted manually based on the outline of cells. The first five frames of each movie were averaged. The averaged image was thresholded (4.5 times the mean intensity of an area with no fluorescent spots) and filtered with a user-defined program in Matlab (MathWorks Corp.). Finally, time courses of the integrated fluorescence intensity of each fluorescent spot were extracted for bleaching analysis.

### Single-Molecule Fluorescence Tracking and Subsequent Analysis.

Time-lapse series of individual ErbB2-EGFP images were acquired to 300 images per sequence at a frame rate of 10 Hz. Detection and tracking of single receptor molecules were performed using a robust tracking program (u-track) (51, 52) as previously described (53). For the calculation of diffusion coefficient, two-dimension MSD was generated for each time interval Δt_n_ of each trajectory, according to the previously described formulas (54):

$$MSD\left(n\delta t\right)=\frac{1}{N-n}\sum_{i=1}^{N-n}\left\{{\left[x\left(i\delta t+n\delta t\right)-x(i\delta t)\right]}^{2}+[y\left(i\delta t+n\delta t\right)-y(i\delta t){]}^{2}\right\},$$

in which, Δt_n_ (Δt_n_ = nδt, with δt = 100 ms) is the elapsed time that a single ErbB2-EGFP molecule move from position x(iδt), y(iδt) to x(iδt + nδt), y(iδt + nδt). n and i are integers. n determines the time increment and takes on values 1, 2, 3 … N-1. N is the total number of image frames. According to the equation MSD_t→0_ = 4DΔt_n_, the diffusion coefficient (D) of each molecule was determined from the slope of the first four points in the MSD-Δt_n_ plot by fitting at least squares principle.

### Statistics Analysis.

Statistical significance was examined using GraphPad Prism software. Data were evaluated to determine whether they conformed to a normal (Gaussian) distribution using Column analyses (Shapiro–Wilk normality test). If the data were normally distributed, unpaired Student’s *t* test was applied for comparison between two groups, and one-way ANOVA followed by Dunnett’s multiple-comparison or Tukey’s multiple-comparison test was applied for comparison between more than two groups. If the data were skewed distribution, a nonparametric test was applied for comparisons. All statistical analyses were two-sided, and statistical significance was evaluated at the *P* < 0.05 level.

## Supplementary Material

Appendix 01 (PDF)

Movie S1. Sample movie of TβRII multiple-step fluorescence quenching.

## Data Availability

All study data are included in the article and/or supporting information.
