# Supplementary material for: A signaling molecule from intratumor bacteria promotes trastuzumab resistance in breast cancer cells
Source: Proc Natl Acad Sci U S A. 2025 Jan 9;122(2):e2421710122. doi: 10.1073/pnas.2421710122 (PMC11745319; doi:10.1073/pnas.2421710122)
Supplement: Supplementary file 1 — Appendix 01 (PDF) [file pnas.2421710122.sapp.pdf]

Supporting Information for

A signaling molecule from intratumor bacteria promotes trastuzumab resistance in breast cancer cells

Gege Qin[a,e], Xiying Shao[d], Xiaolong Liu[a,c], Jiachao Xu[f], Xiaojia Wang[d], Wenxi Wang[b], Lu Gao[b,d], Yuxin Liang[a,c], Lina Xie[d], Dan Su[d], Hongwei Yang[a], Wei Zhou[b,1], and Xiaohong Fang[a,b,c,1]

[1]To whom correspondence may be addressed. Email: zhouwei@him.cas.cn or xfang@iccas.ac.cn

This PDF file includes:

Materials and Methods
Figures S1 to S17
Tables S1 to S8
Legend for Movie S1
SI References

Other supporting materials for this manuscript include the following:

Movie S1

Materials and Methods

Plasmid construction. The DNA fragments encoding full-length TβRII and ErbB2 were respectively subcloned into the HindIII/BamHI and KpnI/SmaI sites of pEGFP-N1 (Clontech), yielding the TβRII-EGFP and ErbB2-EGFP expression plasmids. Plasmid of HA-TβRII was constructed as previously described (1).

Reagents. N-(3-oxo-dodecanoyl) homoserine lactone (3oc) (10007895) was from Cayman Chemical. DMSO (D2650) and Heregulin β1 were from Sigma-Aldrich. Recombinant human TGF-β1 (7754-BH-005) and TGF-β1/2/3 monoclonal antibody (mAb, MAB1835-SP) was from R&D Systems. Trastuzumab was from Roche (Switzerland). LY2109761 (2704) was from Selleck. Annexin V-Alexa Fluor 488/propidium iodide (PI) apoptosis detection kit (FXP022-100) was from 4A Biotech. Cell Counting Kit 8 (CK04) was from DOJINDO. Lipofectamine 3000 (L3000015) was from Thermo Fisher Scientific. For western blots, anti-β-actin mAb (A5316) and anti-TβRII antibody (AV44743) were from Sigma-Aldrich; phospho-ErbB2 (2247), ErbB2 (4290), phospho-PI3 Kinase P85 (4228), phospho-Akt (4060), Akt (4691), phospho-Erk (4370), Erk (4695), phospho-Smad2 (3108) and Smad2 (5339) rabbit mAb were all from Cell Signaling Technology. For immunofluorescence, phospho-Smad2/3 rabbit mAb (8828) was from Cell Signaling Technology. Hoechst 33258 (H21491) and goat anti-rabbit secondary antibody Alexa Fluor Plus 647 (A32733) were from Thermo Fisher Scientific. For flow cytometry, HA Tag mAb DyLight 650 (26183-D650) was from Thermo Fisher Scientific.

Stimulation solution preparation. The 3oc powder was dissolved in DMSO to prepare a 10-20 mM stock solution. For the 3oc treatment group, the stock solution was proportionally diluted into the cell culture medium to achieve the indicated final concentration. The diluted 3oc solution was then added to the cells, which were subsequently incubated under standard conditions for the specified duration. After incubation, the cells were rinsed twice with DPBS to remove any residual treatment solution, followed by the appropriate downstream experimental procedures. In all drug treatment experiments, solvent controls (using similar amounts of solvent) were included.

Cell viability assay. Cell proliferation was detected with the Cell Counting Kit 8 following the manufacturer's instructions. In brief, cells were plated at 1000-6000 cells/well into 96-well plates. Cells were treated for 72 h with 100 μL of culture medium containing a concentration gradient of trastuzumab, either alone or in combination with DMSO, 3oc, or 3oc + LY2109761. Following the treatment, the culture medium was replaced with a fresh solution containing 10% CCK8. The mixture was continuously incubated for 2-4 h at 37 °C, and the absorbance at 450/600 nm was detected by an enzyme-linked immunosorbent assay. Wells with no cells were used as background.

Cell apoptosis was examined using the annexin V/PI apoptosis detection kit. When the cells were stimulated with 3oc or left untreated for 6 h, we performed Annexin V-Alexa Fluor 488 staining for 5 min at room temperature. Subsequently, PI was added to the cells and the apoptotic response was determined immediately by flow cytometry.

For the colony formation assay, cells were seeded at 1000-2000 cells/well into 24-well plates and co-treated with different concentrations of trastuzumab combined with DMSO or 3oc or 3oc + LY2109761 after cell adherence. The culture medium was refreshed every 3 days. Post-growing for 2–3 weeks, the cells were stained with 0.05% crystal violet solution after fixation with 4% paraformaldehyde at room temperature. The dye was washed off, and the colonies were then scanned. ImageJ software package was used for image analysis and colony quantification.

Synergy Analysis. Using the SynergyFinder program (2) (https://synergyfinder.fimm.fi), the anticipated medication combination reactions were computed based on the ZIP reference model, which assumes that the dose-response curves of two non-interacting drugs are expected to show minimal changes. Positive and negative values, calculated as ZIP Synergy scores, representing the deviations between the observed and expected responses, indicate synergy and antagonism, respectively.

RNA isolation, library preparation, and High-Throughput sequencing. BT-474 cells in a 6-well plate were serum starved overnight and then treated with DMSO or 1 μM 3oc for 1 hour. To extract the total RNAs, the cells from each group were washed once with iced PBS and lysed with 1 mL TRIzol reagent (15596-026, Invitrogen Life Technologies). Total RNA was extracted and purified by the miRNeasy Serum/Plasma Advanced Kit (Qiagen, cat. No. 217204) according to the kit protocol. RNA concentration and purity were assessed with the RNA Nano 6000 Assay Kit of the Agilent Bioanalyzer 2100 System (Agilent Technologies, CA, USA). For long-stranded RNA libraries, 250 pg - 10 ng RNA per sample was used as input material, and index codes were added to attribute sequences to each sample. Sequencing libraries were constructed by the SMARTer Stranded Total RNA-Seq Kit V2 (Takara Bio USA, Inc.) according to the manufacturer's recommendation. Finally, library quality was assessed using the Agilent Bioanalyzer 2100 and quantitative real-time PCR. The clustering of the index-coded samples was performed on the acBot Cluster Generation System using TruSeq PE Cluster Kitv3-cBot-HS (Illumina, San Diego, CA, USA) following the manufacturer's protocol. Post cluster generation, the library preparations were sequenced on an Illumina NovaSeq 6000 platform, and paired-end reads were generated.

Differential gene expression and enrichment analyses. The raw data (raw reads) in Fastq format were first processed using in-house Perl scripts. Clean data (clean reads) were acquired in this stage by eliminating adapter-containing reads, unknown base N (unidentifiable base) ≥ 10% containing reads and low-quality reads. Meanwhile, the clean data's Q20/Q30/GC-content, and sequence duplication level were computed. High-quality reads were aligned to the human reference genome (GRCh38) through TopHat2/Bowtie2 software. Gene expression analyses were conducted using Stringtie to compute FPKMs of coding genes in each sample. FPKM indicates fragments per kilo-base of exon per million fragments mapped, calculated based on the length of the fragments and reads count mapped to this fragment. Differential expression analyses were conducted using the DESeq2 R package with cutoff FPKM>5, P-value ≤ 0.02, and Fold change ≥ 1.5 for DEGs.

Reactome (3) and PANTHER (4) pathway enrichment analyses of the DEGs enrichment analyses were performed using the Gene Ontology enrichment analysis tools (5) (http://geneontology.org/). Kyoto Encyclopedia of Genes and Genomes (KEGG) (6) pathway enrichment was implemented using the KEGG Orthology Based Annotation System platform (7) (http://kobas.cbi.pku.edu.cn/). The gene symbol lists of differentially up-regulated and down-regulated genes were submitted to the server for analysis. The data were loaded into R, and dot plots were generated.

Western blotting analysis. Cells grown in a 6-well plate were serum starved overnight and allowed to reach 60%–80% confluence prior to treatment with DMSO, TGF-β or 3oc for a certain period of time in the absence or presence of 0.5 μM LY2109761. Cell extracts were lysed in RIPA buffer containing protease and phosphatase inhibitor cocktails (78442, Thermo Fisher Scientific) for 10 min on ice, immediately spun in a pre-cooled centrifuge (4 ˚C) for 5 min (14,000 rpm) and quantified using BCA Protein Assay Kit (P0010, Beyotime Biotechnology). Equal amounts of protein (10-40 μg) were mixed with non-reducing SDS loading buffer (MB01015, GenScript), boiled at 95 ˚C for 10 min, electrophoresed through 4-20% SDS polyacrylamide gels and were then transferred to polyvinylidene difluoride membranes (IPVH00010, Millipore). The membranes were blocked with 5% non-fat milk (232100, BD) at room temperature for 1 h and then incubated with primary antibodies overnight at 4 ˚C. After thorough washing with TBST (0.05% Tween-20), the membranes were incubated with HRP-labeled secondary antibodies (Beyotime Biotechnology) at room temperature for 1 h followed by three washes with TBST (0.05% Tween-20), and the signals were detected using super enhanced chemiluminescence detection reagent (P1050, Applygen).

Real-time qPCR assay. To check the presence of *Pseudomonas aeruginosa* (*P. aeruginosa*) in breast tumor tissues, we extracted genomic DNA (gDNA) from formalin-fixed paraffin-embedded (FFPE) tissues with FFPE Tissue Genomic DNA One-Step Extraction Kit (RC1004, Concert). gDNA from each specimen was subjected to real-time PCR to search for *P. aeruginosa* presence using designed strain-specific primers targeting the 16S genes. Real-time qPCR reactions were carried out using TaqMan One-Step RT-qPCR Kit (T2210, Solarbio Science & Technology) following the manufacturer's instructions. Amplification and detection of DNA was performed with the Applied

Biosystems 7500 Real-Time PCR System (Thermo Fisher Scientific) under the following reaction conditions: 5 min at 95 ˚C, followed by 40 cycles of denaturation at 95 ˚C for 30 s, at 58 ˚C for 30 s and 72 ˚C for 30 s.

Fluorescent in situ hybridization (FISH). FFPE tissue slides were deparaffinized in 100% xylene for 20 min, fresh 100% xylene for 20 min, then rehydrated in 100% ethanol, fresh 100% ethanol, 95% ethanol, and finally in DEPC water, each for 5 min. Tissue sections were washed in 2×SSC (#AM9765, Ambion), incubated in 1 mg/mL lysozyme (62970, Sigma) for 10 min at room temperature, and then digested with preheated proteinase K (20 μg/ml in 2×SSC, #AM2546, Ambion) for 20 minutes at 37 ˚C. Slides then underwent two washes with 2×SSC followed by two washes in 2×SSC with 30% formamide (#AM9342, Ambion). The probes (listed in Figure S11A) were designed to specifically target the bacterial 16S ribosomal RNA (16S rRNA, EUB338 labeled with Alexa Fluor 488) or specifically *P. aeruginosa* (PseaerA/B labeled with Cy3). The scramble probe labeled with Cy5 (Figure S11A), a ‘‘non-specific probe’’, was used as a FISH negative control. Slides were prehybridized with preheated hybridization buffer containing 30% formamide, 10% dextran sulfate (#D8906, Sigma), 1 mg/mL E.coli tRNA (#R4251, Sigma), 2 mM Vanadyl-ribonucleoside (#S1402S, New England Biolabs), 0.02% BSA (#AM2616, Ambion) and 2× SSC buffer for 2 hours at 41 ˚C. All probes were diluted to 2 μM in preheated hybridization buffer and hybridized to the tissue overnight at 41 ˚C. Sections were then, in turn, washed twice with 2×SSC, twice with 1×SSC, and twice with 0.5×SSC, each for 5 min at 37 ˚C. After staining with 1 ng/mL DAPI (#D9564, Sigma) for 8 min at room temperature, the samples were mounted with Antifade Mounting Medium (#P0126, Beyotime Biotechnology). Images were acquired with the automated slide scanner.

The quantification for *P. aeruginosa* that appeared in breast tumor tissues in scan images was determined by batch processing in TrackMate 7 (Fiji).

Liquid chromatography ionization tandem mass spectrometry (LC-ESI-MS/MS) analysis. Methanol (MeOH) and acetonitrile (ACN) were purchased from Merck (Darmstadt, Germany). MilliQ water (Millipore, Bradford, USA) was used in all experiments. Formic acid was bought from Sigma-Aldrich (St. Louis, MO, USA). Ethyl acetate was purchased from CNW Technologies. The standard stock solution was prepared at a concentration of 1 mg/mL in MeOH and stored at -20 °C. The stock solution was diluted with 50% acetonitrile/water to working solutions before analysis.

After the frozen human breast cancer samples were thawed and smashed, an amount of 0.05 g of the sample was mixed with 500 μL ethyl acetate containing 0.1% formic acid. The sample was vortexed for 5 minutes and centrifuged at 12000 r/min for 10 min at 4 °C. Take 400 μL of supernatant into a new centrifuge tube and concentrate it at 20 °C until completely dry. Then, the sample was redissolved with 100 μL methanol, vortexed for 5 min, and centrifuged at 12000 r/min for 3 min at 4 °C. After centrifugation, 80 μL of the supernatant was analyzed.

The sample extracts were analyzed using an LC-ESI-MS/MS system (UPLC, ExionLC AD, https://sciex.com.cn/, MS, QTRAP® 6500+ System, https://sciex.com/). The analytical conditions were as follows: column, ACQUITY HSS T3 (i.d.2.1×100 mm, 1.8 μm); solvent system, water with 0.1% formic acid (A), methanol with 0.1% formic acid (B); The gradient was started at 5% B (0-1 min), increased to 95% B (5-7 min), finally ramped back to 5% B (7.1-8 min); flow rate, 0.45 mL/min; temperature, 40°C; injection volume, 2 μL.

AB 6500+ QTRAP® LC-MS/MS System, equipped with an ESI Turbo Ion-Spray interface, operating in positive ion modes and controlled by Analyst 1.6 software (AB Sciex). The ESI source operation parameters were as follows: ion source, turbo spray; source temperature, 500 °C; ion spray voltage (IS), 5500 V (Positive); curtain gas (CUR) was set at 20 psi; DP and CE for MRM transitions was done with further DP and CE optimization.

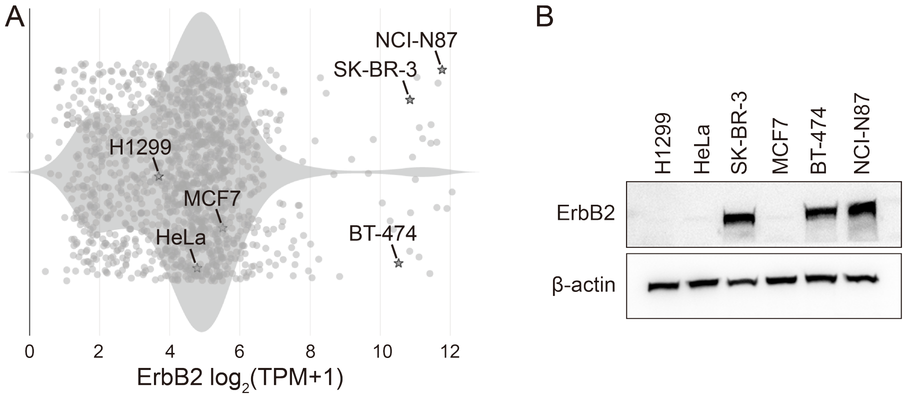

Fig. S1. The ErbB2 expression levels of cancer cells. (A) Characterization of ErbB2 expression levels in several types of cancer cells according to DepMap expression 22Q2 public database (https://depmap.org/portal/data_page/?tab=allData). (B) Western blot was conducted on the cellular expression levels of the above cancer cells. The result indicated that BT-474, SK-BR-3, and NCI-N87 cells endogenously over-express ErbB2, while MCF7, HeLa, and H1299 endogenously low-express ErbB2.

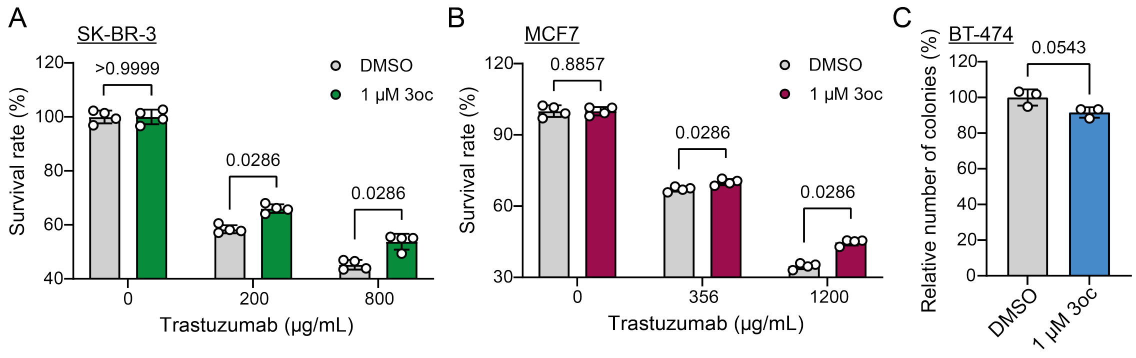

Fig. S2. The cell viability of breast cancer cells treated with trastuzumab and 3oc. (A-B) The dose-response analysis of SK-BR-3 (A) and MCF7 (B) cells to trastuzumab in the presence of 3oc or dimethylsulphoxide (DMSO) control for 72 h. Data are mean ± S.D. n = 4 biological replicates. (C) The colony formation ability of BT-474 cells treated with 3oc or left untreated. Data are mean ± S.D. n = 3 biological replicates. Statistical significance was tested using the two-tailed unpaired Student's *t* test.

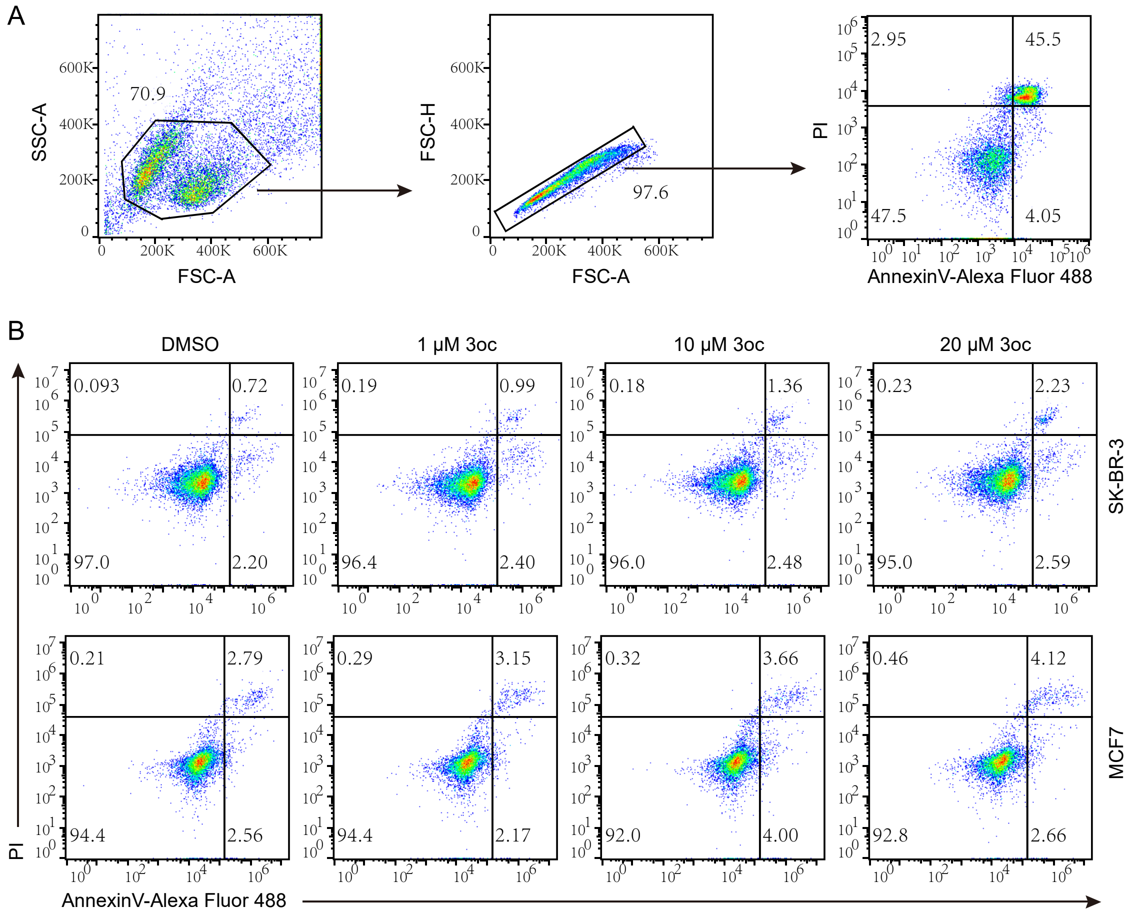

Fig. S3. The apoptosis of breast cancer cells treated with 3oc. (A) Flow cytometric gating strategies for analyses of the cell apoptosis in the presence of 3oc. (B) The dot plot diagram from flow cytometry shows the progression of apoptosis in SK-BR-3 and MCF7 cells exposed to different concentrations of 3oc for 6 h.

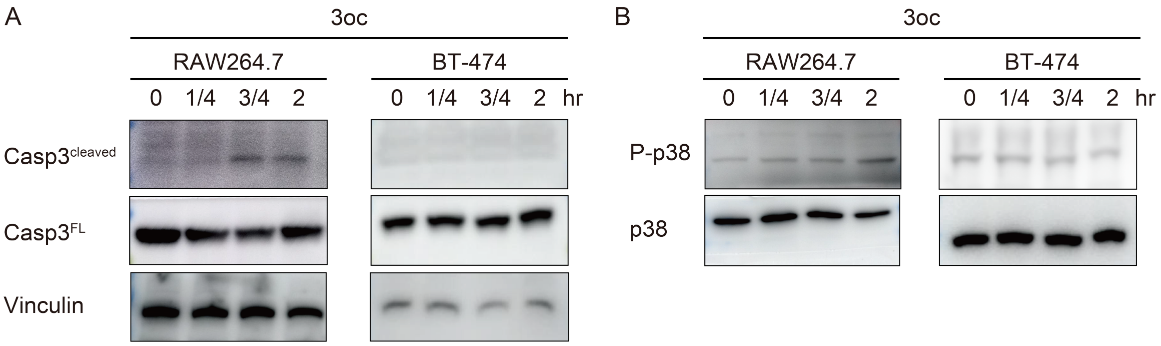

Fig. S4. Western blot analysis for apoptotic caspases (A), p38, and its phosphorylated form (B) in extracts from RAW264.7 and BT-474 cells stimulated with 3oc (10 μM) as indicated.

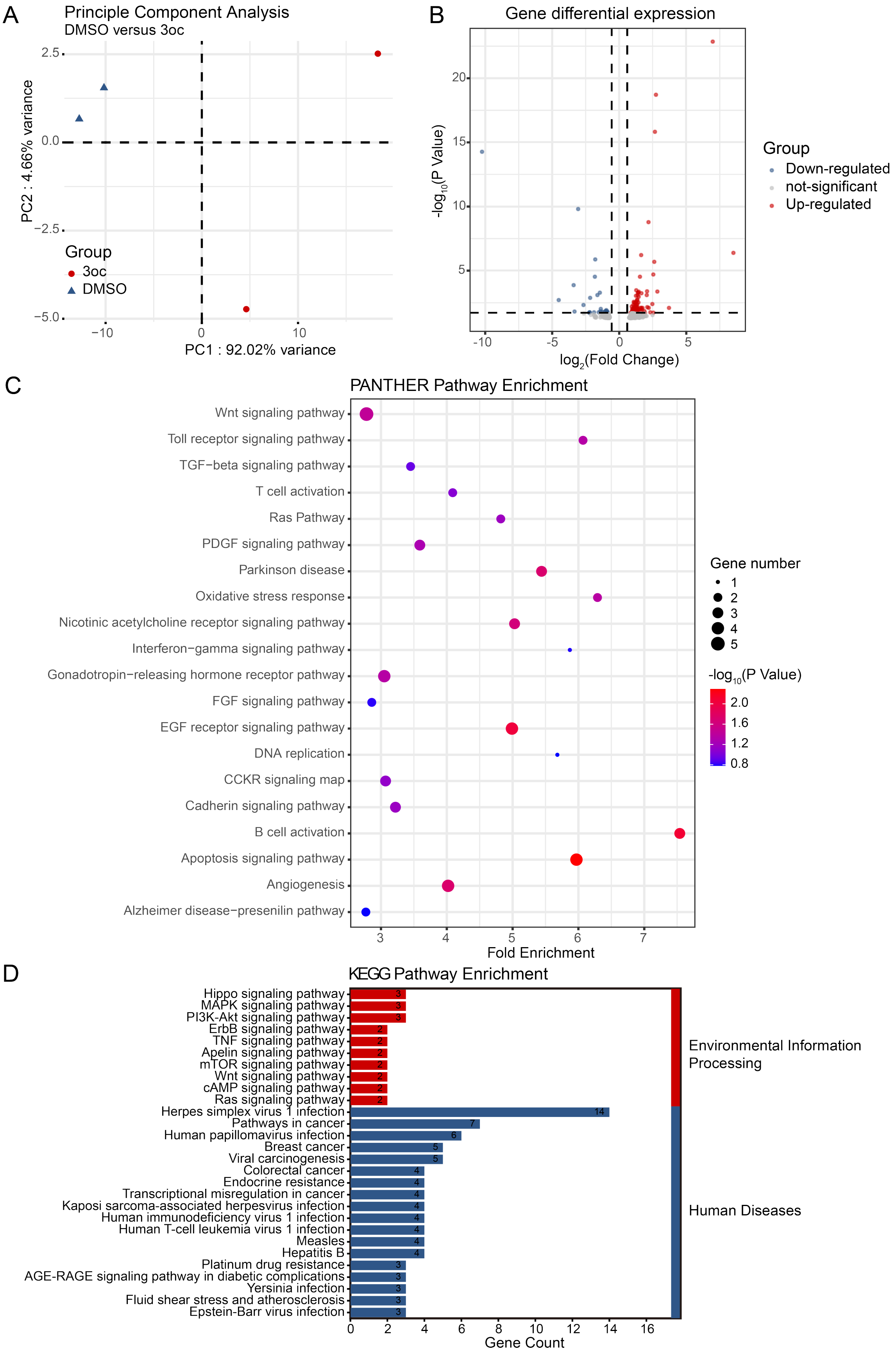
A
Principle Component Analysis
DMSO versus 3oc
PC2 : 4.66% variance
PC1 : 92.02% variance
Group
3oc
DMSO
B
Gene differential expression
-log10(P Value)
log2(Fold Change)
Group
Down-regulated
not-significant
Up-regulated
C
PANTHER Pathway Enrichment
Wnt signaling pathway
Toll receptor signaling pathway
TGF−beta signaling pathway
T cell activation
Ras Pathway
PDGF signaling pathway
Parkinson disease
Oxidative stress response
Nicotinic acetylcholine receptor signaling pathway
Interferon−gamma signaling pathway
Gonadotropin−releasing hormone receptor pathway
FGF signaling pathway
EGF receptor signaling pathway
DNA replication
CCKR signaling map
Cadherin signaling pathway
B cell activation
Apoptosis signaling pathway
Angiogenesis
Alzheimer disease−presenilin pathway
Fold Enrichment
Gene number
-log10(P Value)
D
KEGG Pathway Enrichment
Hippo signaling pathway
MAPK signaling pathway
PI3K-Akt signaling pathway
ErbB signaling pathway
TNF signaling pathway
Apelin signaling pathway
mTOR signaling pathway
Wnt signaling pathway
cAMP signaling pathway
Ras signaling pathway
Herpes simplex virus 1 infection
Pathways in cancer
Human papillomavirus infection
Breast cancer
Viral carcinogenesis
Colorectal cancer
Endocrine resistance
Transcriptional misregulation in cancer
Kaposi sarcoma-associated herpesvirus infection
Human immunodeficiency virus 1 infection
Human T-cell leukemia virus 1 infection
Measles
Hepatitis B
Platinum drug resistance
AGE-RAGE signaling pathway in diabetic complications
Yersinia infection
Fluid shear stress and atherosclerosis
Epstein-Barr virus infection
Gene Count
Environmental Information Processing
Human Diseases

Fig. S5. The mRNA-seq and gene set enrichment analyses of breast cancer cells treated with 3oc. (A) Principal-component analysis (PCA) plot of transcriptomes in BT-474 cells with or without 3oc stimulation from 2 biological replicates. 3oc-treated and control samples are clearly separated. (B) Volcano plot of the 623 differentially expressed genes (DEGs) between the BT-474 cells treated with or without 1 μM 3oc. Each dot indicates a different gene. The genes were filtered for expression changes more significant than 1.5-fold in duplicate arrays with a False Discovery Rate of < 0.02. 163 unique genes that passed the criteria are shown in blue (downregulated) and red (upregulated). (C) Top 20 PANTHER terms associated with differentially translated mRNA targets in BT-474 cells treated with 3oc or left untreated. The complete list of gene set enrichment results and FDR values can be found in supplementary table 5. (D) Kyoto Encyclopedia of Genes and Genomes (KEGG) pathway gene sets enrichment analysis of the DEGs in environmental information processing and human diseases category.

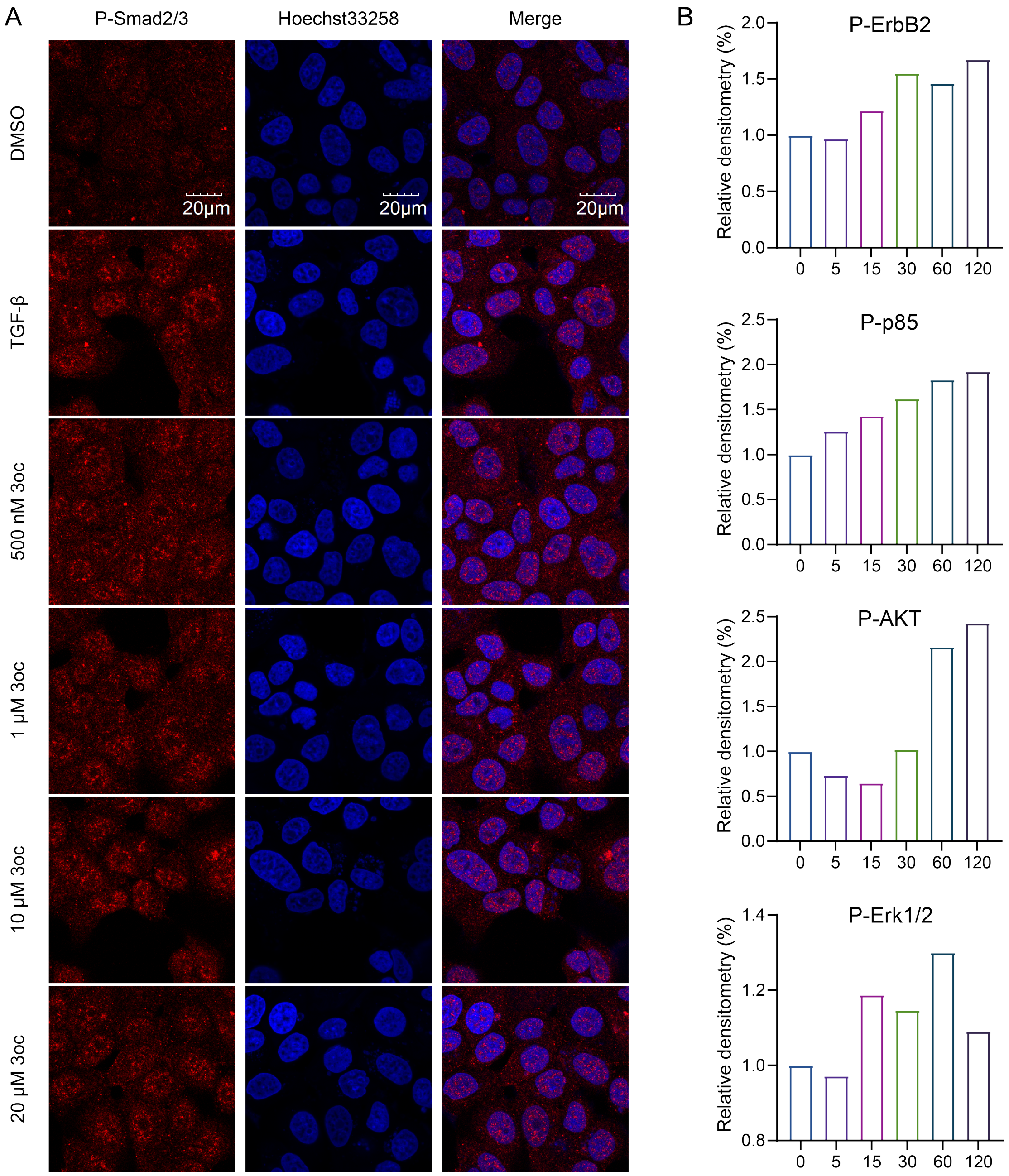

Fig. S6. 3oc activated TGF-β and ErbB2 signaling for breast cancer cells. (A) 3oc induced Smad2/3 phosphorylation and nuclear transportation. Representative immunofluorescence imaging of p-Smad2/3 (mAb, Alexa-647) and cell nuclei (Hoechst 33258) by confocal microscopy in MCF7 cells stimulated with 10 ng/mL TGF-β1 or the indicated concentrations of 3oc. (B) The densitometry analysis of the western blot is in Figure 2D.

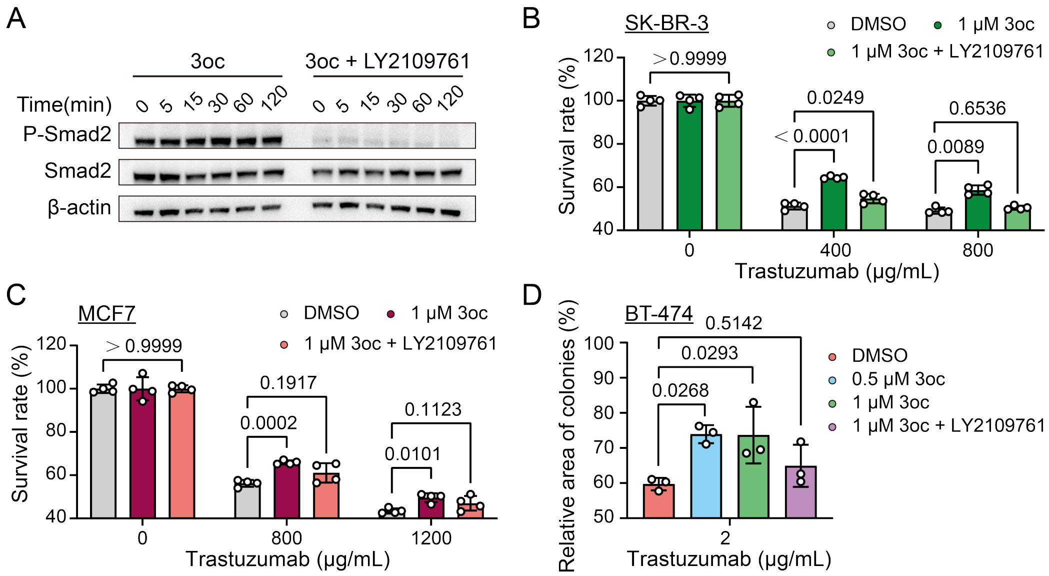

Fig. S7. Effect of LY2109761 on 3oc-induced trastuzumab resistance in breast cancer cells. (A) Immunoblotting of the phosphorylation levels of Smad2 in BT-474 cells stimulated with 3oc (1 μM) alone or the combination of 3oc (1 μM) and LY2109761 (0.5 μM) in time gradient. (B-C) The dose-response analysis of SK-BR-3 cells (B) and MCF7 cells (C) to trastuzumab in the treatment of indicated compounds for 72 h. Data are the mean ± S.D. n = 4 biological replicates. (D) The quantitation of colony formation ability of BT-474 cells treated with the indicated concentrations of trastuzumab in the presence or absence of 3oc or 3oc + LY2109761 (0.5 μM). Colony-forming units were determined for each treatment group. The overall *P* value is 0.0272. Data are the mean ± S.D. n = 3 biological replicates. Statistical significance was tested by one-way ANOVA with Dunnett's multiple comparisons test.

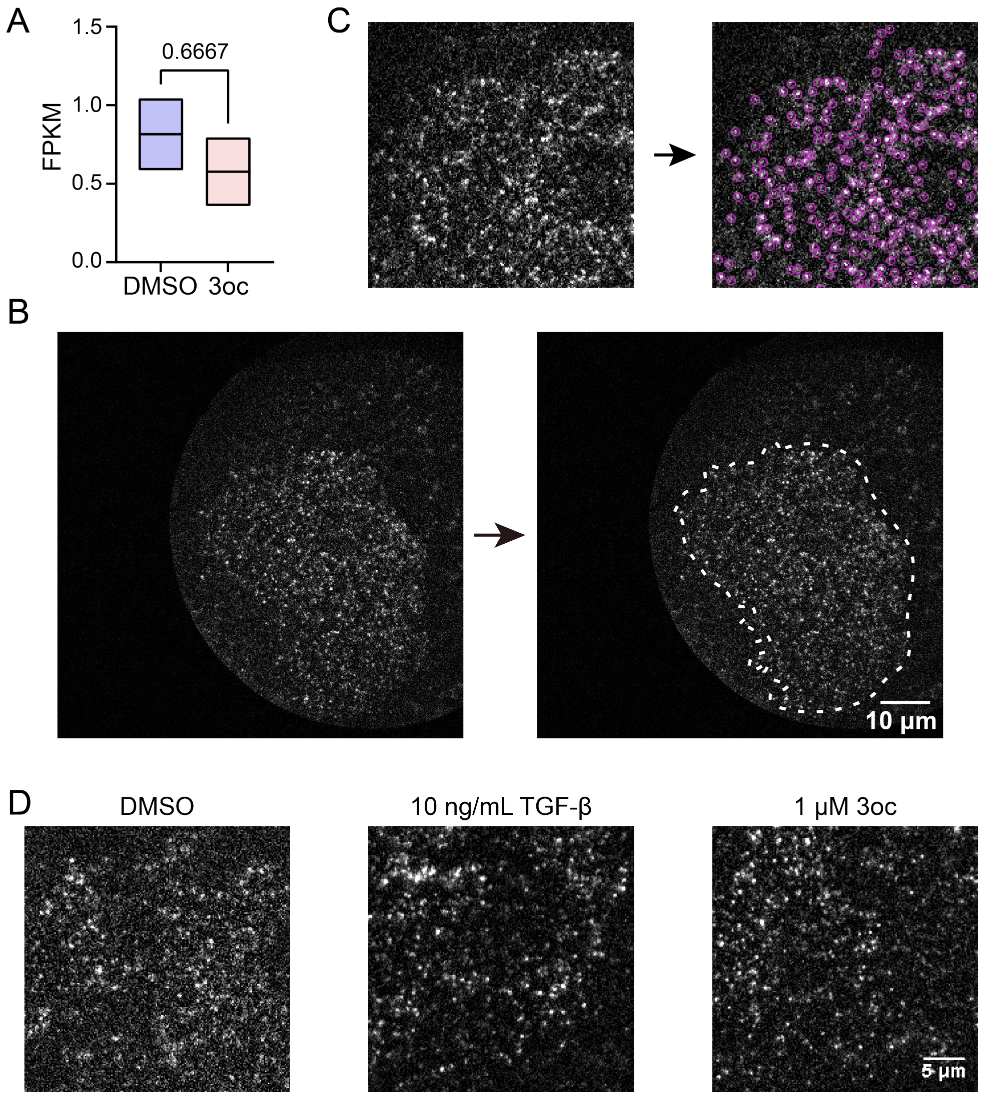

Fig. S8. 3oc directly induced the spontaneous dimerization of TβRII on the cell membrane without external ligand. (A) The Fragments Per Kilobase of transcript per Million fragments mapped (FPKM) histogram of transcripts of TGF-β1 (ENSG00000105329) in BT-474 cells treated with 3oc (1 µM) or left untreated. Boxplots display values of minimum, median, and maximum. Non-parametric Mann–Whitney test. (B) A representative total internal reflection fluorescence microscope (TIRFM) image of one tumor cell transfected with TβRII-EGFP plasmids. The cell contour was outlined with white dashed lines. (C) A typical single-molecule image (left panel) shows the density and size of TβRII-EGFP molecules at the plasma membrane of a MCF7 cell imaged with TIRFM. The image is a section (32×32 µm) of the averaged first five frames from a raw movie, and then the background was subtracted. The diffraction-limited spots (5×5 pixel regions) enclosed with purple circles represented the signals from individual TβRII-EGFP puncta (right panel) and were chosen for intensity analysis. (D) Typical images showing the individual TβRII-EGFP molecules docking on the MCF7 cell membrane stimulated with TGF-β 1 or 3oc.

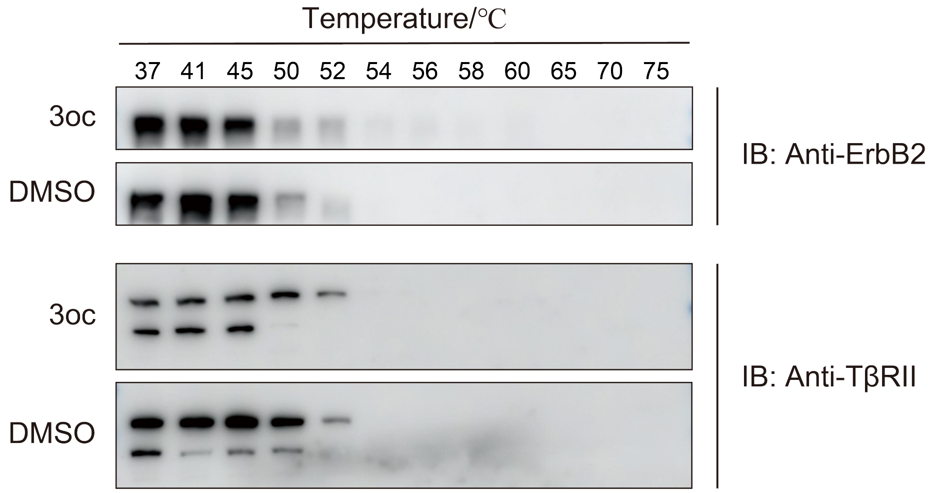

Fig. S9. Western blot analysis of ErbB2 and TβRII from Cellular Thermal Shift Assay in BT-474 cells pretreated with 10 μM 3oc.

A

Target gene	Sequence (5’-3’)
Primers	
gyrB	AACAAGGTCTGGGAACAGGTCTAC<br>CATCGGTCTCGCCCACTTC
β-globin	CCAAGAGTCTTCTCTGTCTC<br>GAAGTTGGTGGTGAGGCC
Probes	
gyrB	TAMRA - CCACGGCGTTCCGCAGTTCC - BHQ2
β-globin	FAM - AGGTTGGTATCAAGGTTACAAG - MGB

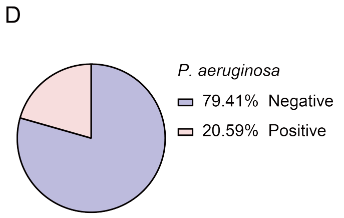

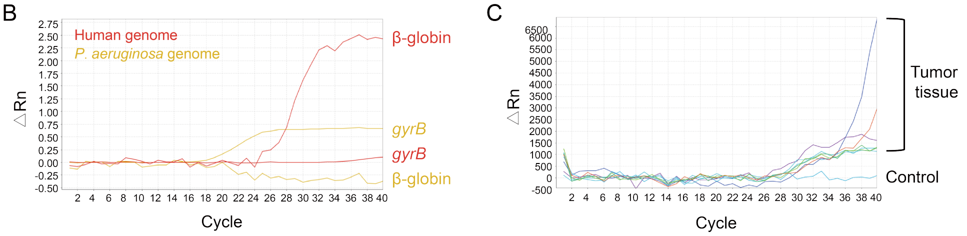

Fig. S10. The detection of *P. aeruginosa* in clinical breast tumor tissues through qPCR. (A) Sequences of primers and probes of the target genes. (B) Amplification curves of *gyrB* and β-globin target genes, using genomic DNA extracted from *P. aeruginosa* and human tissues as templates. (C) Amplification curves of the *gyrB* target gene, using the breast cancer tissues or negative controls as templates. (D) The pie chart shows the proportion of *P. aeruginosa* positive and negative detections in breast cancer specimens.

A

Probe	Target species	Probe sequence (5'-3')
EUB338	Eubacteria	[Alexa488] GCTGCCTCCCGTAGGAGT
PseaerA	*P. aeruginosa*	[Cy3] GGTAACCGTCCCCCTTGC
PseaerB	*P. aeruginosa*	[Cy3] TCTCGGCCTTGAAACCCC
Scramble	Non-specific	[Cy5] CGACGGAGGGCATCCTCA

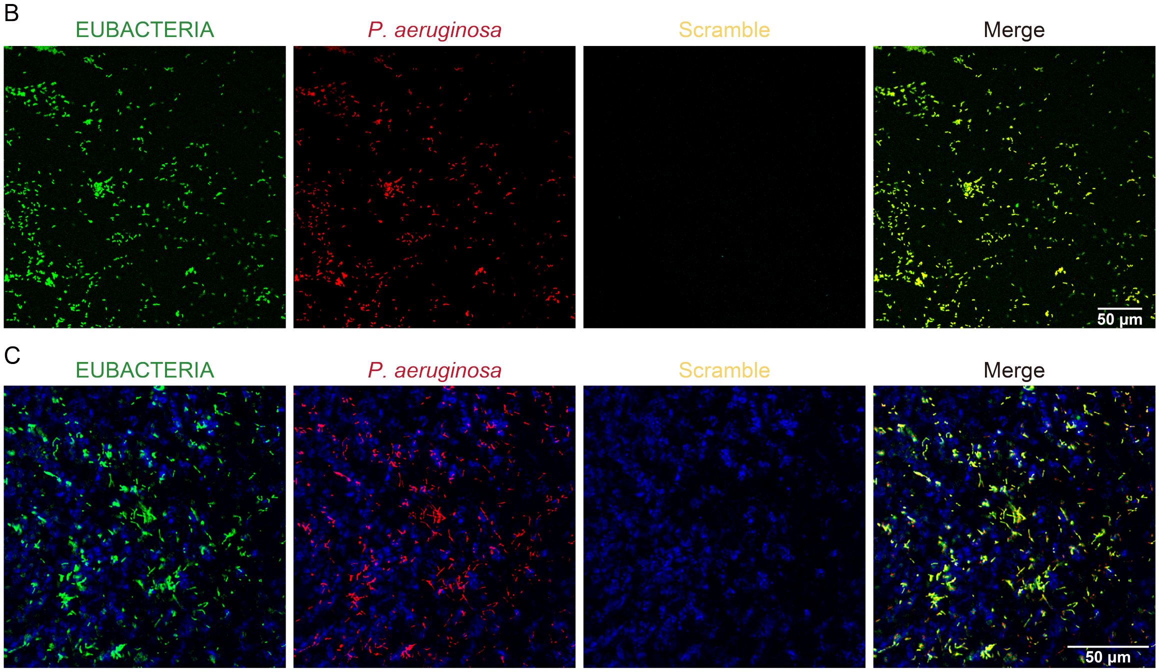

Fig. S11. Detection of *P. aeruginosa* in clinical breast tumor tissues through fluorescence in situ hybridization (FISH) imaging. (A) FISH probes for *P. aeruginosa* detection. FISH staining for *P. aeruginosa* in glass slides coated with *P. aeruginosa* (B) and mouse mammary tumors injected intratumorally with *P. aeruginosa* (C). Samples were stained with eubacteria probes (green), *P. aeruginosa* probes (red), and scramble probes (yellow). The FISH images demonstrated co-labeling of *P. aeruginosa* by the EUB338 and PseaerA/B probes, which produced the characteristic rod-like morphology of *P. aeruginosa*. Meanwhile, the Scramble probe channel showed no specific fluorescence signal, confirming the specific binding of the PseaerA/B probe to *P. aeruginosa*. Furthermore, *P. aeruginosa* signals were more intensive in pronounced tissue necrosis. These results indicated that our probe system can be used to detect *P. aeruginosa* in tumor tissues.

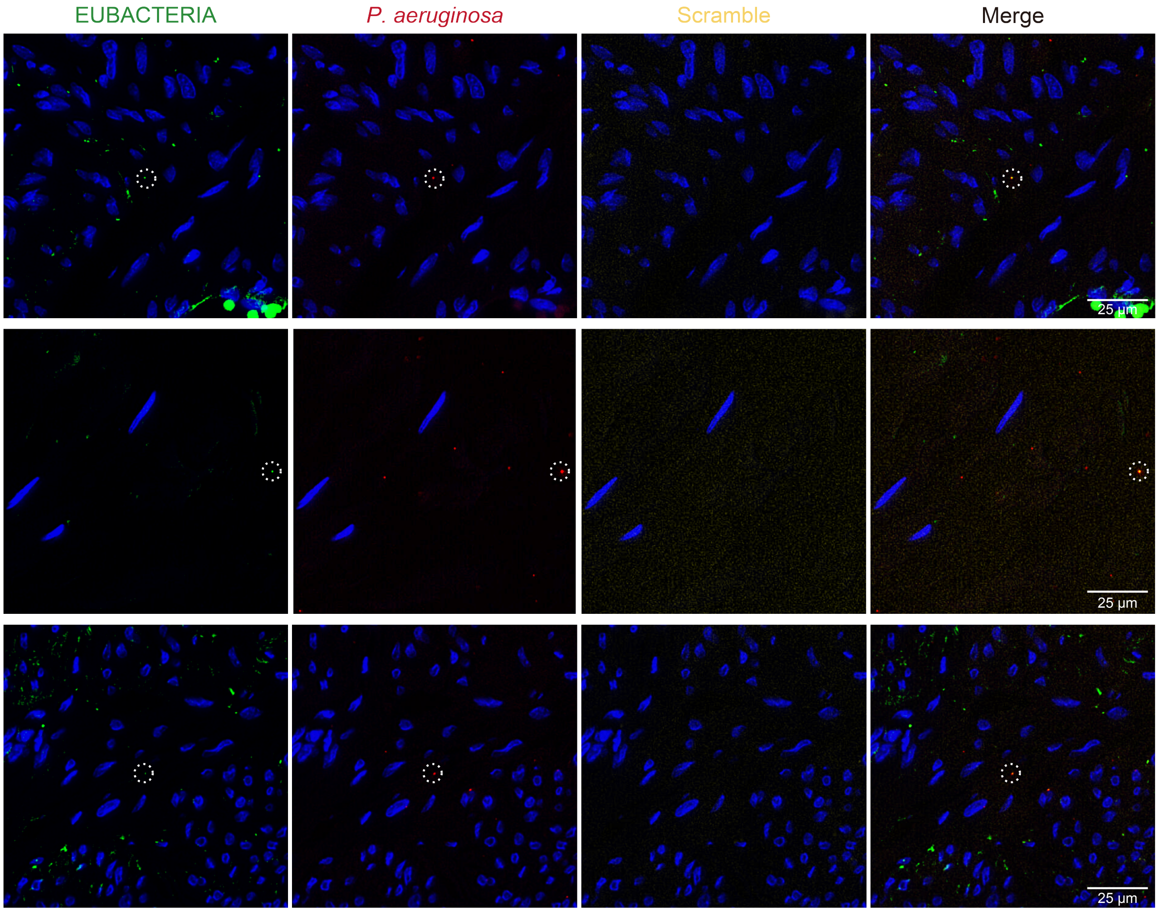

Fig. S12. FISH staining images of *P. aeruginosa* in three human breast tumor samples. *P. aeruginosa* are encircled by white dashed circles.

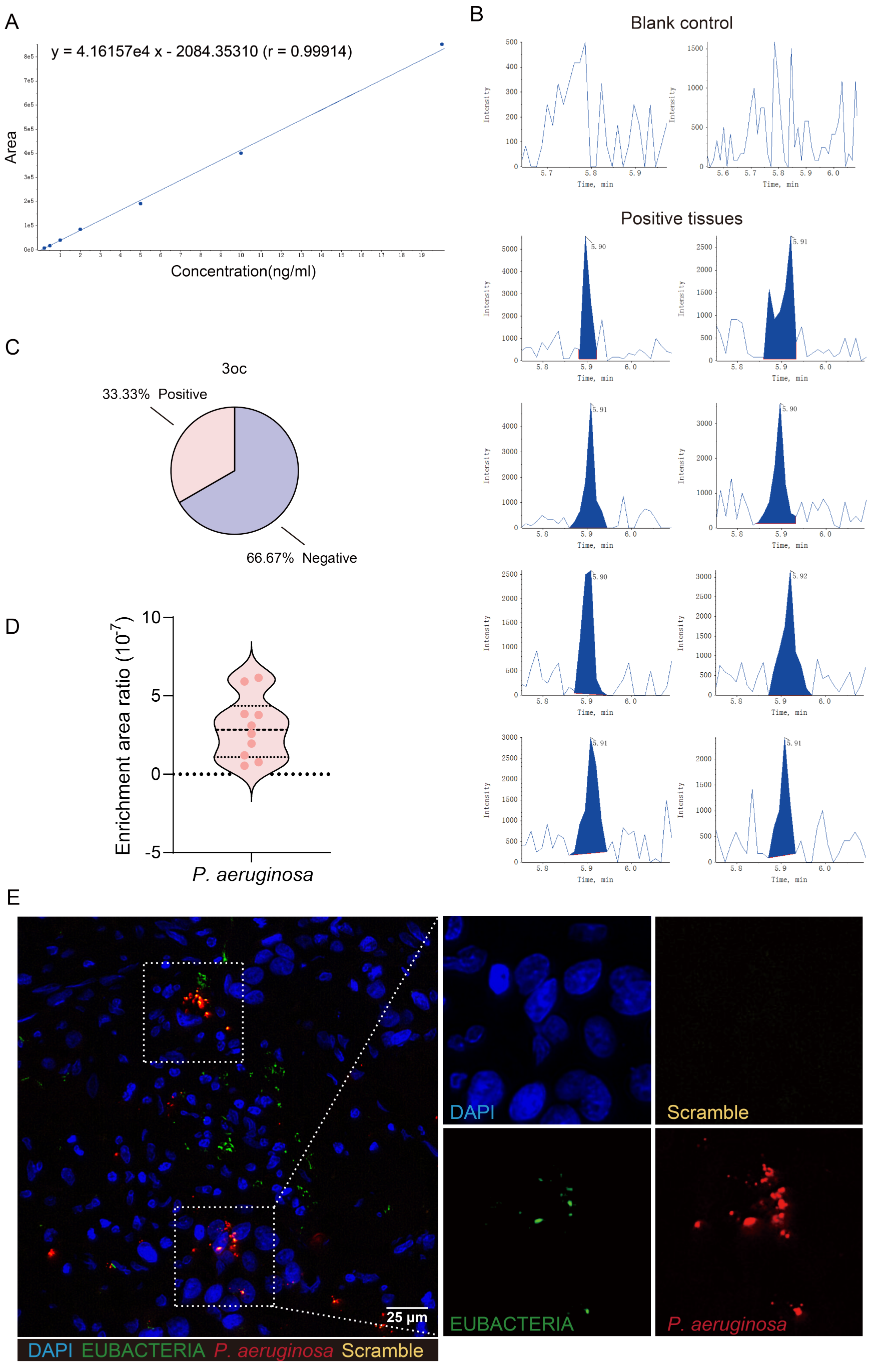

Fig. S13. Detection of 3oc and *P. aeruginosa* in clinical breast tumor tissues. (A) Quantitative standard curve for 3oc by mass spectrometry. (B) Substance integration diagram for 3oc detection assay in frozen tumor samples from breast cancer patients. (C) The pie chart for the proportion of 3oc positive and negative detections in breast cancer specimens. (D) The proportion of *P. aeruginosa*-enriched area relative to whole tissue volume. n=10 *P. aeruginosa*-enriched areas. (E) FISH staining images for *P. aeruginosa* in one human breast tumor sample. *P. aeruginosa* enriched areas are encircled by white dashed rectangles. A boxed region is enlarged and shown. Based on the analysis of multiple regions within tumor tissues enriched with *P. aeruginosa*, the volume occupied by these bacterial colonies constitutes approximately 10^{-7} of the total tumor tissue volume. This suggests that the concentration of *P. aeruginosa*'s secreted signaling molecule, 3oc, may be similarly enriched within these regions, potentially reaching micromolar levels, with an estimated enrichment factor of 10^{7}.

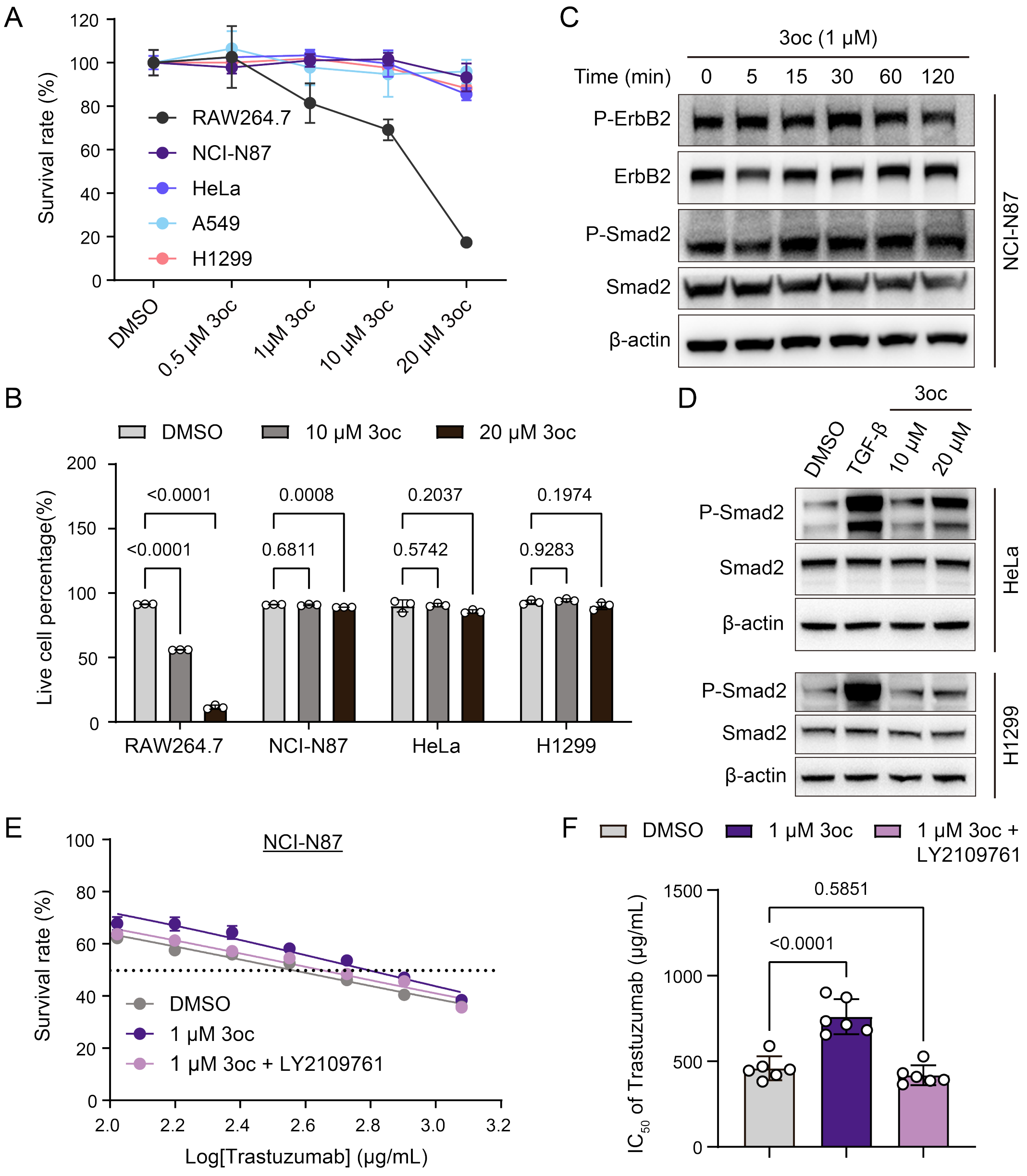

Fig. S14. Effect of 3oc on gastric, cervical, and lung cancer cells. (A) Cell proliferation was tested on different types of tumor cells co-cultured with increasing concentrations of 3oc (0.5–20 μM) or left untreated for 72 h. Data are the mean ± S.D. n = 6 biological replicates. (B) Cell apoptosis was detected by flow cytometry on the above cell types after treatment with increasing concentrations of 3oc for 6 h. Data are the mean ± S.D. n = 3 biological replicates. (C) Representative western blot of the phosphorylation levels of Smad2 and ErbB2 in NCI-N87 cells stimulated with 1 μM 3oc in time gradient. (D) Representative western blot of the phosphorylation levels of Smad2 in HeLa and H1299 cells stimulated with the different concentrations of 3oc for 30 min. (E) The dose-response analysis of NCI-N87 cells to trastuzumab (0–1200 μg/mL) in the presence or absence of 3oc or 3oc + LY2109761 (0.5 μM) for 72h. (F) The quantitative data showed the IC_{50} to trastuzumab for each treatment group. The overall *P* value is <0.0001. Data are the mean ± S.D. n = 6 biological replicates. Image is representative of at least 2 independent experiments (C, D). Statistical significance was tested by one-way ANOVA with Dunnett's multiple comparisons test.

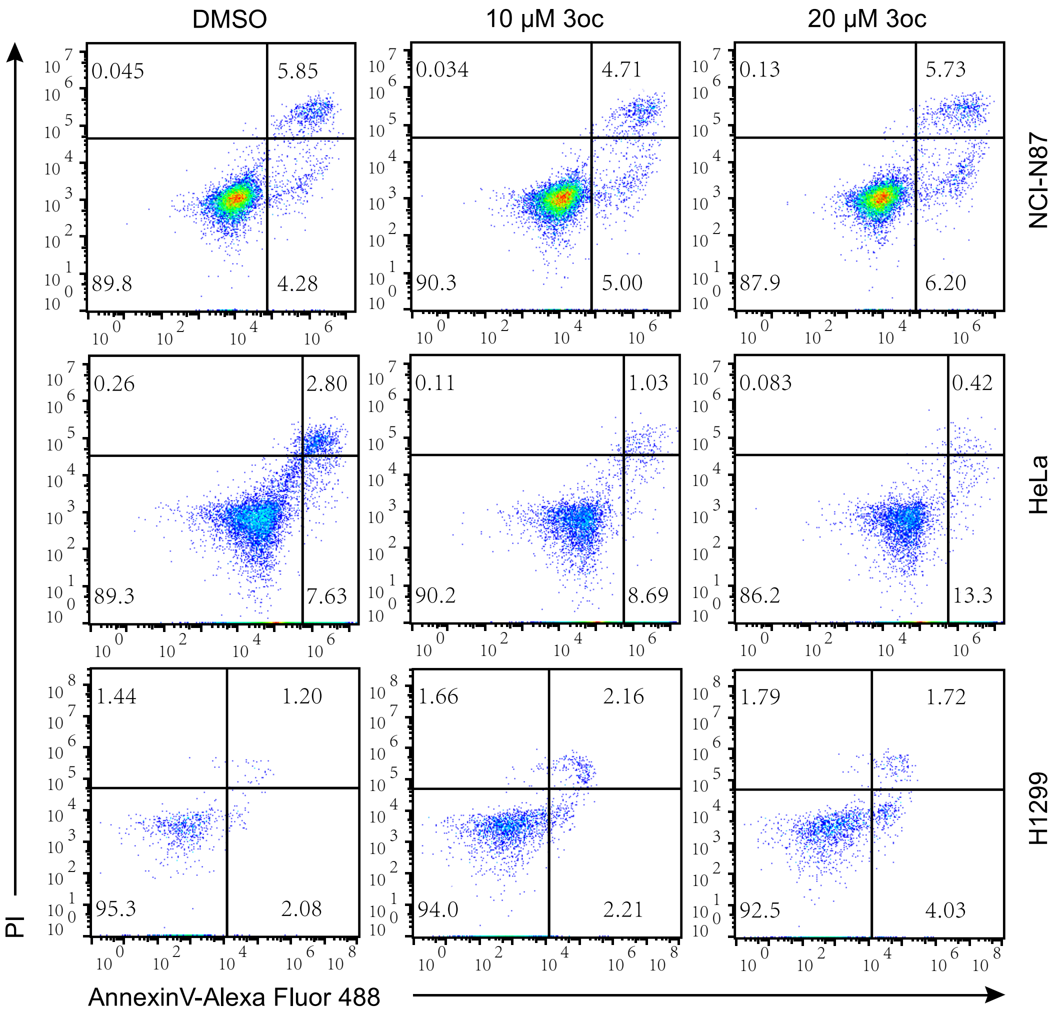

Fig. S15. The apoptosis of several cancer cells treated with 3oc. The dot plot diagram from flow cytometry showed the progression of apoptosis in NCI-N87, HeLa and H1299 cells exposed to a 3oc concentration gradient for 6 h.

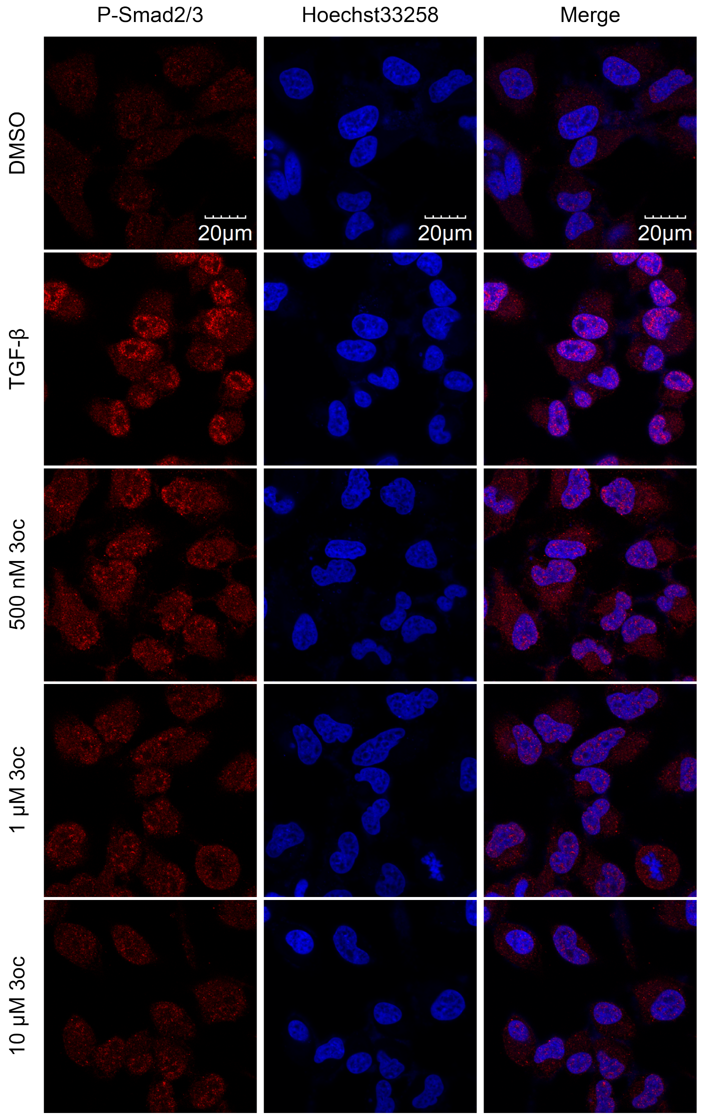

Fig. S16. 3oc induced Smad2/3 phosphorylation and nuclear transportation. Immunofluorescence imaging of p-Smad2/3 (mAb, Alexa-647) and cell nuclei (Hoechst 33258) by confocal microscopy in HeLa cells stimulated with 10 ng/mL TGF-β1 or the indicated concentrations of 3oc.

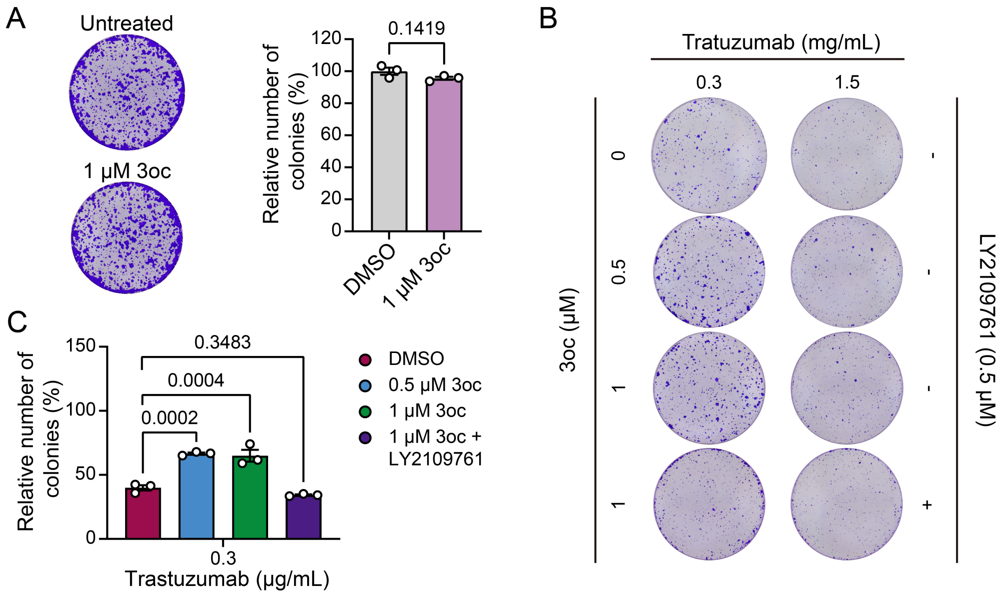

Fig. S17. Effect of LY2109761 on 3oc-induced trastuzumab resistance in gastric cells. (A-B) Representative colony formation images of BT-474 cells treated with the indicated concentrations of trastuzumab in the presence or absence of 3oc or 3oc + LY2109761. (C) Colony-forming units were determined for each treatment group. The overall *P* value is <0.0001. Data are the mean ± S.D. n = 3 biological replicates. Statistical significance was tested by two-tailed unpaired Student's *t* test (A) and one-way ANOVA with Dunnett's multiple comparisons test (C).

Table S1. Significant differential-expressed genes after 3oc treatment in BT-474 cells, Related to Figure 2

Gene_id	DMSO1 FPKM	DMSO2 FPKM	3oc1 FPKM	3oc2 FPKM	FDR	Log2FC
80757	0	0	0.565194	0.613554	4.10E-07	8.516548
3310	0.065488	0.01093	7.302479	4.868394	1.40E-23	6.974983
123879	0.074297	0.055864	0.159446	1.883391	0.0079992	3.711663
79820	1.106942	1.606729	2.457894	10.46654	0.000425	2.84386
57561	4.325719	4.603225	26.54263	41.34926	1.94E-19	2.739921
1958	2.045283	1.94842	15.37503	12.99637	1.50E-16	2.662809
387522	2.734483	3.243872	8.349569	13.79002	2.05E-06	2.613345
6248	0.140411	0.183885	0.399462	1.837034	0.0187676	2.548273
11080	0.464727	0.584544	2.670349	4.309176	1.99E-05	2.54257
140564	0.126229	0.108693	0.799684	0.73421	0.0039989	2.469714
222662	0.316934	0.193048	0.518836	1.53995	0.0176132	2.321599
100533105	0.813706	0.323963	2.982773	2.102762	0.0079221	2.20138
57786	1.086192	0.809088	4.846218	4.863964	1.65E-09	2.182687
100526842	23.805965	27.989678	44.30467	231.0274	0.006968	2.091043
79973	0.369407	0.825295	2.070363	3.338043	0.0004061	2.076393
126068	0.510296	0.58282	2.016059	3.503878	0.0007983	2.052934
285331	0.267327	0.325817	1.018729	1.575672	0.0193684	1.921844
2353	0.571617	0.583684	2.435627	2.21783	0.0140946	1.874284
79637	1.485657	1.715561	5.181937	4.49837	0.0079992	1.739868
153222	0.603307	0.808204	1.156072	1.686914	0.0109515	1.701074
5366	2.771799	3.054638	9.128821	11.65372	0.0005749	1.655406
163051	0.39213	0.53562	1.204541	1.788134	0.0197816	1.650827
85459	0.572009	0.529998	1.40173	2.292677	0.0079221	1.650353
9782	4.190175	3.373936	12.42366	12.82268	6.09E-07	1.635894
84077	0.51992	0.394256	1.432851	1.694233	0.0079221	1.596408
7738	0.949299	0.983594	2.829568	3.774835	0.0122489	1.578107
5926	0.676971	0.852426	1.824471	2.676846	0.0105173	1.570072
284370	0.725415	0.846338	2.238227	3.181504	0.0197816	1.552462
3725	11.038443	9.199456	39.4312	26.59902	2.97E-05	1.546002
54813	0.79305	1.057588	2.090243	4.005917	0.0118824	1.519039
360023	2.170426	2.798107	5.669054	9.989582	0.0004353	1.456108
26973	2.492844	2.800366	5.277785	8.41768	0.0010791	1.452312
114803	1.331942	1.649387	3.171284	5.805428	0.0029957	1.432337
374860	1.130119	2.072285	3.397696	5.291684	0.0173615	1.42469
163050	3.049849	3.46217	8.345734	11.3068	0.0005201	1.410392
7644	0.984877	1.433179	3.241071	3.941887	0.0141864	1.404291
55632	2.715182	2.702202	6.145304	10.40848	0.0005201	1.401214
55015	3.567673	3.68455	7.795843	12.02467	0.0008179	1.377334
84146	1.601208	1.505073	3.465834	5.775599	0.0061591	1.350843
79101	7.560485	7.933193	15.64952	27.56469	0.0017828	1.349018
55279	1.110983	1.133203	2.273957	4.248369	0.0158837	1.346594

114799	1.831748	1.820121	4.11118	6.171007	0.0039095	1.341125
23036	0.7438	0.842442	1.622133	3.036383	0.0079992	1.328701
84081	2.461126	2.384624	5.56548	7.685628	0.0019963	1.327095
57082	1.018183	1.051949	2.095297	3.864301	0.0079992	1.324362
23253	0.882701	0.734296	1.746466	2.771848	0.0120475	1.319703
339500	1.196613	1.840759	3.387374	5.480535	0.0077879	1.307728
4820	1.143473	1.212095	2.457732	4.339386	0.0115311	1.305374
6782	1.649899	2.879384	4.816737	7.837842	0.0197447	1.301577
7516	3.140215	3.214102	6.426584	11.32012	0.0025579	1.298546
375444	2.627517	3.309395	6.26801	9.968992	0.01411	1.297284
4189	3.239643	3.918632	8.154539	11.64124	0.0079992	1.288101
79596	2.567624	2.867601	6.225056	8.665782	0.0044057	1.283774
79982	1.831493	1.985078	3.224451	6.654551	0.0175401	1.279437
9988	2.736057	4.102275	6.060963	11.66302	0.0114273	1.275307
284391	4.846887	5.172671	11.28197	16.1348	0.0003359	1.269787
546	1.221995	1.5552	2.839291	4.930194	0.0093116	1.256868
9994	1.089654	1.268536	2.410307	3.767156	0.0173615	1.256244
23325	1.812334	2.811831	4.589582	8.012752	0.0154969	1.252541
253260	1.159448	1.390753	2.872346	4.499357	0.0109515	1.249957
221037	2.405294	2.167713	3.656818	6.140226	0.0062743	1.247774
65117	6.573281	7.801407	16.95673	23.357	0.0009077	1.245732
51633	2.439116	3.043836	6.0151	8.418519	0.012871	1.242957
55183	1.256711	1.539955	2.773674	5.399634	0.0079992	1.242825
55320	1.893901	2.220335	4.301936	6.842323	0.0093231	1.241629
55023	1.750743	1.97908	3.73286	6.303296	0.0061149	1.24044
10224	2.453742	2.602552	5.882039	7.575466	0.0176132	1.235617
8204	2.034975	2.28418	4.136796	7.103715	0.0064453	1.225664
9360	2.739015	3.066568	5.464254	10.06565	0.007316	1.219232
160418	1.419707	1.508969	2.629767	5.127585	0.0193284	1.21692
259266	3.169433	2.915904	5.385847	9.974031	0.0066407	1.213339
163059	4.048027	4.392408	10.07891	11.99491	0.0140946	1.206873
286826	2.303856	2.976316	5.298105	7.87387	0.0138698	1.202438
1195	9.099244	9.579033	19.8378	29.9805	0.0030681	1.199988
94104	3.089134	3.994912	6.582746	10.87234	0.0079992	1.198353
26046	1.358553	1.689313	2.86259	5.489391	0.016319	1.194492
22894	1.559527	1.809994	3.361711	5.672411	0.0110443	1.187222
375743	1.283066	1.558479	2.43343	4.755307	0.0173615	1.185849
64786	1.714717	2.043541	3.847187	5.643665	0.0107802	1.183992
55892	2.590865	2.621714	5.29982	7.963515	0.004368	1.183785
9958	1.499872	1.394456	2.702024	3.505474	0.0067837	1.178695
80124	1.326967	1.73552	2.908471	4.945924	0.0114273	1.175647
168451	3.39656	3.412406	7.042199	10.40143	0.0087202	1.173308
51710	2.271198	2.73209	4.914421	8.517944	0.0103945	1.171229
140890	2.129315	2.796705	3.694684	6.710608	0.0136307	1.165259
389677	2.233712	2.252199	3.944391	6.112603	0.0073688	1.163954

9669	4.972418	5.245671	9.641257	16.02842	0.007316	1.144408
57695	2.814772	3.450254	6.286814	9.201784	0.0061591	1.141465
134266	3.019554	4.174146	7.185766	10.48054	0.0115311	1.129246
22847	1.547455	1.711501	3.084512	4.855328	0.0141864	1.129138
26512	2.109927	2.741361	4.654868	7.095414	0.0087927	1.125444
25788	3.658062	4.238508	7.184233	11.76224	0.0193284	1.113975
5874	6.567977	8.314074	13.66398	22.89049	0.0087364	1.112508
51742	5.611594	6.357711	14.76365	16.61528	0.0030681	1.112205
10142	2.175579	2.497112	3.80478	6.285445	0.0193284	1.112035
5876	8.840023	8.765949	14.13326	23.57778	0.0114273	1.106156
55074	4.197581	5.056572	8.198765	13.26859	0.0141864	1.106043
10773	5.750954	7.003506	11.98648	18.97386	0.0096511	1.104206
51174	8.977349	11.075399	16.73744	29.4308	0.007882	1.103289
394	1.8398	2.224111	3.856143	5.947831	0.011429	1.089355
645455	3.802414	4.689382	8.143664	11.88308	0.0079992	1.088855
29110	4.217684	5.035495	9.044778	13.04263	0.0141864	1.084928
199692	7.58006	9.327252	18.09646	21.40402	0.0026808	1.083842
54014	0.998575	1.339346	3.089164	4.190867	0.0175401	1.079965
23469	0.760244	0.91316	1.504925	2.298294	0.0173615	1.078754
83540	6.665739	7.487938	14.1621	19.43994	0.0140946	1.077215
51068	6.562134	7.851329	11.06124	24.88742	0.0124297	1.073588
8036	3.825633	4.952336	7.889753	12.98408	0.0179907	1.063033
339745	4.019701	5.351666	8.123353	13.74105	0.0197985	1.060311
23347	2.942049	4.036642	6.41345	10.15258	0.0187676	1.060033
10592	1.637075	1.824721	3.109465	4.954128	0.0193284	1.057515
23350	6.13095	7.296837	11.74295	19.99417	0.0172985	1.048739
51747	19.119233	19.605117	35.54526	51.07247	0.0047498	1.047522
113251	4.128415	4.924613	7.955208	13.43373	0.0197816	1.042997
5033	4.458163	5.832108	9.725551	11.48877	0.0153431	1.041035
905	3.699402	3.654049	6.710543	10.32396	0.0197816	1.017901
6873	5.714571	6.184749	11.36327	13.89756	0.0105305	1.017566
79752	8.464772	7.887085	11.19733	18.77573	0.0193684	1.008639
51602	12.09467	15.317255	27.47726	36.68624	0.0115311	1.00811
10051	9.766513	13.367855	20.43331	31.77715	0.0197816	1.001
8821	4.867203	5.265585	8.643886	12.97247	0.0173615	0.98931
10403	7.111445	9.356326	15.80684	20.67142	0.0193284	0.989064
7572	14.089621	17.320384	25.98962	44.39014	0.0181615	0.982132
55660	6.693646	8.169747	12.7654	21.08621	0.0197985	0.980024
163049	2.821622	4.064114	6.674686	9.361387	0.0182268	0.978984
54443	16.670278	19.634179	32.63262	50.91601	0.0153818	0.978113
58517	5.289789	5.680536	8.495229	14.45957	0.0193684	0.975331
9735	4.331564	4.956786	8.010529	11.46319	0.0172985	0.971016
1316	10.076833	11.329748	20.96372	26.10743	0.0095448	0.967393
7153	11.918479	14.547578	23.11579	35.40815	0.0138545	0.964849
904	4.360888	4.786434	8.194558	11.99339	0.0197816	0.962019

57532	6.083328	7.210571	11.10381	18.12758	0.0193099	0.945948
10512	8.018935	8.940606	14.66175	22.0076	0.0197985	0.942858
3320	201.077859	227.843096	390.0965	538.4784	0.0062328	0.938986
1894	15.237556	18.872617	28.76253	45.15978	0.0197603	0.93427
10808	18.63426	22.753901	36.23385	53.49316	0.0173607	0.924869
55602	9.258639	10.383504	18.28223	23.54366	0.0197603	0.918026
7227	7.962994	9.255877	14.4113	21.80134	0.0197447	0.90026
9584	37.921432	36.990596	60.22665	101.5473	0.0190464	0.859933
6712	12.775392	11.629285	10.17059	7.032706	0.0155118	0.873352
29	21.183105	21.841422	17.25284	16.62807	0.0182163	0.884838
7916	77.067825	67.322045	48.92327	38.16846	0.0175401	-0.89219
4854	29.565532	27.126919	18.85452	14.12655	0.0145928	0.950237
5339	42.462757	34.60022	25.09945	19.85452	0.0118824	0.960117
1026	13.724581	12.153498	7.041656	7.698265	0.0197447	0.989148
1277	17.688769	15.660913	10.04146	7.986788	0.0140946	1.004545
26173	40.949641	35.344813	25.22142	16.78609	0.0140946	1.006404
8675	27.426524	18.296002	13.29637	12.13438	0.0172985	-1.01171
23513	35.370726	28.570317	20.59768	11.89003	0.0173615	-1.10452
80097	107.909891	66.111247	51.0806	33.72849	0.0193284	1.206458
1396	44.048384	26.015104	17.94858	13.03004	0.0158006	1.261843
389816	71.939796	43.452114	31.32586	18.30819	0.0095537	1.375428
100529063	23.379907	9.603422	5.986306	4.097588	0.0148585	1.402685
286262	12.401734	17.655068	6.682791	5.133683	0.0005201	1.443764
388564	72.377243	37.884922	29.04669	13.52066	0.0173615	1.527101
59274	6.274546	9.665016	3.643852	2.089036	0.0007983	1.627453
7108	20.935739	32.569191	8.863647	8.920945	1.33E-06	1.788651
55072	7.645605	6.531925	1.99784	3.007317	2.97E-05	1.816396
8344	1.031996	0.949903	0.343851	0.236586	0.0176469	1.880035
8325	7.798471	5.611522	2.47705	0.819551	0.0013175	2.169669
56917	0.636115	0.630985	0.153101	0.127998	0.0174562	2.202538
84914	0.793196	4.107365	0.492154	0.701451	0.0179907	2.215446
4839	2.732303	0.956221	0.792463	0.3366	0.0190464	2.233171
2738	2.199358	1.830635	0.119619	0.526584	0.0047498	2.666827
440672	6.397543	9.347721	1.064265	1.036751	1.57E-10	3.064301
441518	0.652938	8.107956	0.59506	0.359532	0.0153888	3.333181
126374	0.13885	0.20727	0.026188	0.008751	0.0001344	3.400394
3430	118.984865	5.029679	3.975421	3.718948	0.0019527	4.513597
642636	0.63221	0.411514	0	0	5.39E-15	-10.2333

Table S2. Top 20 terms in that the differential expressed genes after 3oc treatment were enriched in Reactome database, Related to Figure 2

Reactome pathways	Gene count	Fold enrichment	P-value	FDR
Drug-mediated inhibition of ERBB2 signaling	2	88.01	4.68E-04	8.90E-02
Resistance of ERBB2 KD mutants to trastuzumab/lapatinib/AEE788/afatinib/osimertinib/neratinib/tesevatinib/sapitinib	2	88.01	4.68E-04	1.07E-01
Estrogen-dependent gene expression	5	7.46	6.71E-04	1.09E-01
Activation of the AP-1 family of transcription factors	3	52.81	4.82E-05	1.10E-01
TP53 Regulates Transcription of DNA Repair Genes	4	10.83	6.30E-04	1.11E-01
Scavenging by Class F Receptors	2	58.68	8.66E-04	1.24E-01
MAPK targets/ Nuclear events mediated by MAP kinases	3	17.03	9.25E-04	1.24E-01
Constitutive Signaling by Overexpressed ERBB2	2	39.12	1.68E-03	1.83E-01
NR1H2 and NR1H3-mediated signaling	3	11.74	2.53E-03	2.06E-01
TRIF(TICAM1)-mediated TLR4 signaling	4	7.33	2.50E-03	2.20E-01
Transcriptional Regulation by TP53	9	4.4	2.50E-04	2.85E-01
Regulation of PTEN gene transcription	3	8.95	5.21E-03	3.84E-01
Regulation of HSF1-mediated heat shock response	3	7.77	7.60E-03	4.96E-01
Signaling by ERBB2 KD Mutants	2	16.76	7.41E-03	4.98E-01
RNA polymerase II transcribes snRNA genes	3	7.14	9.49E-03	5.16E-01
Signaling by TGF-beta Receptor Complex	3	7.33	8.83E-03	5.17E-01
Transcription of the HIV genome	3	7.23	9.16E-03	5.23E-01
RUNX2 regulates osteoblast differentiation	2	15.31	8.72E-03	5.24E-01
Interleukin-17 signaling	3	7.44	8.51E-03	5.25E-01
VEGFR2 mediated vascular permeability	2	13.04	1.16E-02	5.90E-01
RNA Polymerase II Pre-transcription Events	3	6.29	1.32E-02	5.91E-01
Signaling by Interleukins	7	2.76	1.46E-02	5.97E-01
Defective BTD causes biotidinase deficiency	1	100	1.13E-02	5.98E-01
PIP3 activates AKT signaling	5	3.55	1.45E-02	6.02E-01

Table S3. Top 20 terms in that the differential expressed genes after 3oc treatment were enriched in PANTHER database, Related to Figure S5

PANTHER_pathways	Gene_count	Fold_enrichment	P_value	FDR
Apoptosis signaling pathway	4	5.97	5.09E-03	8.50E-01
B cell activation	3	7.54	8.20E-03	6.85E-01
EGF receptor signaling pathway	4	4.99	9.28E-03	5.17E-01
Angiogenesis	4	4.02	1.88E-02	6.28E-01
Parkinson disease	3	5.44	1.91E-02	5.32E-01
Nicotinic acetylcholine receptor signaling pathway	3	5.03	2.34E-02	5.57E-01
Wnt signaling pathway	5	2.78	3.61E-02	7.53E-01
Oxidative stress response	2	6.29	4.26E-02	7.90E-01
Gonadotropin-releasing hormone receptor pathway	4	3.05	4.44E-02	7.42E-01
Toll receptor signaling pathway	2	6.07	4.53E-02	6.87E-01
PDGF signaling pathway	3	3.59	5.33E-02	7.42E-01
Ras Pathway	2	4.82	6.73E-02	8.64E-01
Cadherin signaling pathway	3	3.22	6.89E-02	8.22E-01
CCKR signaling map	3	3.07	7.69E-02	8.56E-01
T cell activation	2	4.09	8.86E-02	9.24E-01
TGF-beta signaling pathway	2	3.45	1.17E-01	1.00E+00
FGF signaling pathway	2	2.86	1.57E-01	1.00E+00
Interferon-gamma signaling pathway	1	5.87	1.61E-01	1.00E+00
Alzheimer disease-presenilin pathway	2	2.77	1.65E-01	1.00E+00
DNA replication	1	5.68	1.66E-01	1.00E+00

Table S4. Top 10 terms in that the differential expressed genes after 3oc treatment were enriched in environmental information processing and human diseases category of KEGG database, respectively, Related to Figure S5

KEGG_pathways	Gene Count
Herpes simplex virus 1 infection	14
Pathways in cancer	7
Human papillomavirus infection	6
Breast cancer	5
Viral carcinogenesis	5
Colorectal cancer	4
Endocrine resistance	4
Transcriptional misregulation in cancer	4
Kaposi sarcoma-associated herpesvirus infection	4
Human immunodeficiency virus 1 infection	4
Human T-cell leukemia virus 1 infection	4
Measles	4
Hippo signaling pathway	3
MAPK signaling pathway	3
PI3K-Akt signaling pathway	3
ErbB signaling pathway	2
TNF signaling pathway	2
Apelin signaling pathway	2
mTOR signaling pathway	2
Wnt signaling pathway	2
cAMP signaling pathway	2
Ras signaling pathway	2

Table S5. Single sample gene set enrichment analysis of cellular mRNA abundance in MAPK gene sets after 3oc treatment, Related to Figure 2

Gene_symbol	DMSO1_FPKM	DMSO2_FPKM	3oc1_FPKM	3oc2_FPKM
MKNK2	29.214237	23.247823	17.931641	12.822968
STK3	5.397195	6.370133	9.567095	14.166496
FOS	0.571617	0.583684	2.435627	2.21783
HSPA6	0.065488	0.01093	7.302479	4.868394
JUN	11.038443	9.199456	39.431202	26.599022
FLNA	130.947731	113.68637	91.043264	67.899121

Table S6. Single sample gene set enrichment analysis of cellular mRNA abundance in PI3K/Akt gene sets after 3oc treatment, Related to Figure 2

Gene_symbol	DMSO1_FPKM	DMSO2_FPKM	3oc1_FPKM	3oc2_FPKM
KITLG	1.723115	2.476593	3.688185	6.14245
HSP90AA1	201.0779	227.8431	390.0965	538.4784
COL1A1	17.68877	15.66091	10.04146	7.986788
PRLR	14.91432	19.50372	24.68973	40.51161
CREB1	2.417498	3.086255	4.573888	6.812959
LAMA5	11.3829	9.287476	6.999496	5.021257
LPAR6	0.498756	0.586514	1.450751	2.042172
C8orf44-SGK3	0.813706	0.323963	2.982773	2.102762

Table S7. Clinical information on Cohort for qPCR analysis (paraffin tissues), Related to Figure 5A

Patients	Age (years old)	Gender	Pathological type	AJCC stage
1	57	Female	Invasive carcinoma	IV
2	41	Female	Invasive carcinoma	III
3	69	Female	Invasive carcinoma	III
4	37	Female	Invasive carcinoma	IV
5	34	Female	Invasive carcinoma	IV
6	25	Female	Invasive carcinoma	III
7	61	Female	Invasive carcinoma	IV
8	56	Female	Invasive carcinoma	IV
9	54	Female	Invasive carcinoma	IV
10	49	Female	Invasive carcinoma	IV
11	57	Female	Invasive carcinoma	IV
12	63	Female	Invasive carcinoma	IV
13	70	Female	Invasive carcinoma	IV
14	39	Female	Invasive carcinoma	IV
15	51	Female	Invasive carcinoma	IV
16	46	Female	Invasive carcinoma	IV
17	54	Female	Invasive carcinoma	IV
18	63	Female	Invasive carcinoma	IV
19	34	Female	Invasive carcinoma	IV
20	41	Female	Invasive carcinoma	IV
21	42	Female	Invasive carcinoma	IV
22	56	Female	Invasive carcinoma	IV
23	60	Female	Invasive carcinoma	IV
24	33	Female	Invasive carcinoma	IV
25	70	Female	Invasive carcinoma	IV
26	37	Female	Invasive carcinoma	IV
27	54	Female	Invasive carcinoma	IV
28	64	Female	Invasive carcinoma	IV
29	52	Female	Invasive carcinoma	IV
30	56	Female	Invasive carcinoma	IV
31	50	Female	Invasive carcinoma	Unclear
32	42	Female	Invasive carcinoma	IV
33	62	Male	Invasive carcinoma	Unclear
34	57	Female	Invasive carcinoma	IV

Table S8. Clinical information on Cohort for FISH analysis (paraffin tissues), Related to Figure 5C

Patients	Age (years old)	Gender	ErbB2	AJCC stage	pCR	Recurrence	DFS (months)
1	47	Female	+++	II	Yes	No	58.63
2	54	Female	+++	III	No	No	92.83
3	53	Female	+++	II	No	No	64.23
4	50	Female	+++	II	Yes	No	19.77
5	54	Female	+++	II	Yes	No	52.10
6	39	Female	+++	II	Yes	No	69.03
7	29	Female	+++	II	Yes	No	8.43
8	48	Female	+++	II	No	No	5.53
9	55	Female	+++	II	Yes	No	63.00
10	54	Female	+++	III	Yes	No	73.30
11	44	Female	+++	II	Yes	No	0.00
12	67	Female	+++	II	No	No	15.20
13	50	Female	+++	II	No	No	61.30
14	49	Female	+++	II	No	No	30.00
15	55	Female	+++	II	Yes	No	58.83
16	44	Female	+++	II	No	Yes	6.30
17	55	Female	+++	II	No	No	55.97
18	40	Female	+++	II	No	No	61.63
19	49	Female	+++	II	Yes	No	59.77
20	51	Female	+++	II	No	No	51.13
21	29	Female	+++	II	No	No	0.00
22	40	Female	+++	III	No	Yes	8.70
23	61	Female	+++	II	No	Yes	16.77
24	54	Female	+++	II	No	No	50.07
25	53	Female	+++	II	Yes	No	48.33
26	50	Female	+++	II	Yes	No	48.80
27	49	Female	+++	IV	Yes	No	46.93
28	44	Female	+++	II	Yes	No	24.80
29	41	Female	+++	II	No	No	45.60
30	41	Female	+++	II	No	No	43.27
31	55	Female	++	III	No	No	43.50
32	41	Female	+++	II	No	No	37.37
33	34	Female	+++	III	No	Yes	3.83
34	47	Female	+++	II	No	Yes	7.57

Movie S1 (separate file). Sample movie of TβRII multiple-step fluorescence quenching.

SI References

1. M. Cheng *et al.*, Single-molecule dynamics of site-specific labeled transforming growth factor type II receptors on living cells. *Chemical Communications* **50**, 14724-14727 (2014).
2. A. Ianevski, A. K. Giri, T. Aittokallio, SynergyFinder 3.0: an interactive analysis and consensus interpretation of multi-drug synergies across multiple samples. *Nucleic Acids Research* **50**, W739-W743 (2022).
3. M. Gillespie *et al.*, The reactome pathway knowledgebase 2022. *Nucleic Acids Research* **50**, D687-D692 (2022).
4. P. D. Thomas *et al.*, PANTHER: Making genome-scale phylogenetics accessible to all. *Protein Science* **31**, 8-22 (2022).
5. H. Mi, A. Muruganujan, J. T. Casagrande, P. D. Thomas, Large-scale gene function analysis with the PANTHER classification system. *Nature Protocols* **8**, 1551-1566 (2013).
6. M. Kanehisa, S. Goto, KEGG: Kyoto Encyclopedia of Genes and Genomes. *Nucleic Acids Research* **28**, 27-30 (2000).
7. D. Bu *et al.*, KOBAS-i: intelligent prioritization and exploratory visualization of biological functions for gene enrichment analysis. *Nucleic Acids Research* **49**, W317-W325 (2021).
